# A biosensor-based framework to measure latent proteostasis capacity

**DOI:** 10.1038/s41467-017-02562-5

**Published:** 2018-01-18

**Authors:** Rebecca J. Wood, Angelique R. Ormsby, Mona Radwan, Dezerae Cox, Abhishek Sharma, Tobias Vöpel, Simon Ebbinghaus, Mikael Oliveberg, Gavin E. Reid, Alex Dickson, Danny M. Hatters

**Affiliations:** 1 Department of Biochemistry and Molecular Biology, Bio21 Molecular Science and Biotechnology Institute, University of Melbourne, Parkville, VIC 3010 Australia; 2 Department of Physical Chemistry II, Ruhr-University Bochum, Universitaetsstraße 150, 44780 Bochum, Germany; 3 Department of Biochemistry and Biophysics, Arrhenius Laboratories of Natural Sciences, Stockholm University, 10691 Stockholm, Sweden; 4 School of Chemistry, University of Melbourne, Parkville, VIC 3010 Australia; 5 Department of Biochemistry & Molecular Biology, Michigan State University, East Lansing, MI 48824 USA; 6 Department of Computational Mathematics, Science and Engineering, Michigan State University, East Lansing, MI 48824 USA

## Abstract

The pool of quality control proteins (QC) that maintains protein-folding homeostasis (proteostasis) is dynamic but can become depleted in human disease. A challenge has been in quantitatively defining the depth of the QC pool. With a new biosensor, flow cytometry-based methods and mathematical modeling we measure the QC capacity to act as holdases and suppress biosensor aggregation. The biosensor system comprises a series of barnase kernels with differing folding stability that engage primarily with HSP70 and HSP90 family proteins. Conditions of proteostasis stimulation and stress alter QC holdase activity and aggregation rates. The method reveals the HSP70 chaperone cycle to be rate limited by HSP70 holdase activity under normal conditions, but this is overcome by increasing levels of the BAG1 nucleotide exchange factor to HSPA1A or activation of the heat shock gene cluster by HSF1 overexpression. This scheme opens new paths for biosensors of disease and proteostasis systems.

## Abstract (web summary)

A pool of quality control proteins (QC) maintains the protein-folding homeostasis in the cell, but its quantitative analysis is challenging. Here the authors develop a FRET sensor based on the protein barnase, able to quantify QC holdase activity and its ability to suppress protein aggregation.

## Introduction

Protein-folding homeostasis (proteostasis) in humans is controlled by a quality control (QC) network of about 800 proteins^1^. Key cogs of the QC network are the chaperones, such as HSP70 and HSP90 family members, which monitor the foldedness of proteins almost from the moment they emerge from the ribosome and thereon throughout their lifespan. While the QC network can dynamically respond to stresses to maintain proteostasis, it can also become depleted in protein misfolding diseases^1–3^. A challenge has been to define the buffering depth of the QC network in managing proteostasis and to track how it changes when stimulated or challenged.

To understand baseline proteostasis buffering capacity requires the development of new quantitative approaches. Prior schemes have used aggregation of ectopically expressed conformationally destabilized (i.e., metastable) proteins as flags for when proteostasis had declined^4–6^. These schemes operated on the principle that QC systems actively suppress the aggregation rates of metastable “bait” proteins and hence when proteostasis was depleted cells lost the capacity to suppress aggregation. Others have also examined the in-cell folding rates of a test protein through rapid temperature jumps to follow rates for reestablishment of equilibrium^7^. However, these approaches lack a quantitative capacity to understanding proteostasis; namely through the inability to define the effectiveness of QC systems to engage with the bait proteins.

Our motivation was to develop a new biosensor system modeled on these prior schemes—but with a substantially improved quantitative capacity. Here, we describe a biosensor system based on a series of metastable bait proteins that report on foldedness and aggregation state by fluorescence resonance energy transfer (FRET). We show that we can measure the engagement of QC (in terms of the net holdase activity from many individual components) to the unfolded state of the biosensor and concomitant influences on the biosensor aggregation. We describe a new mathematical framework that can extract quantitative information from the holdase activity of QC as well as ability to suppress aggregation. These approaches provide insight to the depth of the pool of QC resources that regulate proteostasis.

## Results

### Barnase as a sensing kernel for a new tunable biosensor

**Fig. 1:**
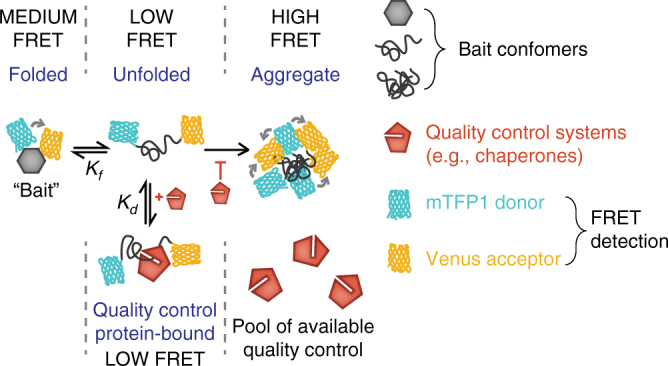
Design strategy for probing proteostasis efficiency. Scheme of how the bait biosensor module measures collective cellular chaperone engagement by binding to unfolded barnase and in preventing aggregation. Medium FRET and low FRET: binding of quality control machinery to barnase bait pulls the equilibrium away from folded barnase, reducing the FRET signal in the soluble pool. High FRET: quality control systems reduce aggregate accumulation, thereby reducing the number of cells with high-FRET

The prior biosensor schemes using metastable proteins typically display complex folding mechanisms, which make good substrates for QC but pose great challenges in mechanistically quantifying the effects in terms of the thermodynamics of the system^4–6^. We hence chose a bait protein through which we could more deeply examine the thermodynamics of protein folding and aggregation in the context of QC engagement. Barnase (in a catalytically inactive form (H102A mutant^8^)–defined hereon as wild-type* based on established nomenclature), was chosen as our bait because it can be predictably tuned to different free energies of folding (Δ*G*_*F*_) by mutation^9–11^. Knowledge of, and the capacity to predictably alter Δ*G*_*F*_, provides a strategy to alter the dynamic range of foldedness and hence provide a handle for more control over defining the extent to which proteostasis alters the folded state of barnase away from thermodynamic equilibrium and basal aggregation levels (Fig. 1).

**Fig. 2:**
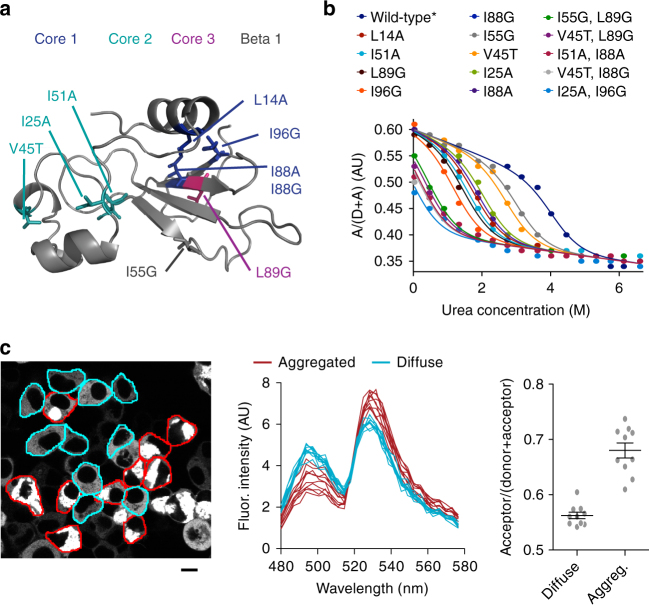
FRET reports on barnase foldedness and aggregation. **a** Bait protein barnase structure is shown (PDB ID 1A2P) with the location of destabilizing mutations used to tune *K*_*f*_ (and thus Δ*G*_*F*_). **b** Urea denaturation curves, assayed by FRET, are shown of barnase constructs expressed in mammalian lysate fitted to a two-state unfolding model (one representative replicate per mutant of *n* = 3). **c** A confocal micrograph image of representative cells expressing a destabilized barnase variant (I25A, I96G) in HEK293T cells. Scale bar=10 µm. The wild-type barnase variant does not form visible aggregates. The middle graph shows fluorescence spectra (excitation 405 nm) for cells with only diffuse biosensor vs. cells with visible aggregates. The right graph shows a proxy measure for FRET of these cells (means ± SEM)

We predicted that a FRET strategy—flanking the barnase moiety with two fluorescent proteins—could be used to monitor barnase conformations; including chaperone-unfolded client complex (Fig. 1). To make the biosensor, we first screened a mini-library of circularly permuted variants of monomeric teal fluorescent protein 1 (mTFP1) and Venus to identify the optimal combination for tracking barnase foldedness (Supplementary Fig. 1). Upon determining the best combination (mTFP1 cp175-barnase-Venus cp173), we made 15 mutants predicted to span a range of equilibrium constants of folding (*K*_*f*_), corresponding to Δ*G*_*F*_ values between −25 kJ/mol (most stable) to 1 kJ/mol (least stable)^10,11^. Denaturation curves of these constructs in purified form or directly in mammalian lysates yielded Δ*G*_*F*_ values that correlated with the previously recorded or predicted Δ*G*_*F*_ values^10,11^, demonstrating that the FRET scheme authentically reports on barnase folding equilibrium (Fig. 2a, b; Supplementary Fig. 2a, b; Supplementary Table 1). Furthermore, assessment of FRET in cells expressing the mutant biosensor revealed the cells containing extensively aggregated biosensor have higher FRET signal than cells lacking aggregates, suggesting that aggregation can be detected as a higher FRET state than the folded and unfolded states (Fig. 2c). Hence, these data confirm the capacity to follow the three FRET states as depicted in Fig. 1: high-FRET (aggregated), medium-FRET (folded), and low-FRET (unfolded).

Next we assessed whether barnase was indeed an appropriate client for sampling mammalian QC. Binding partners to the biosensor were measured by quantitative proteomics on immunoprecipitates of a barnase biosensor tuned to be folded (wild-type*) vs. a variant tuned to be substantially unfolded (I25A, I96G). A variety of heat shock family proteins bound abundantly to both forms of the biosensor based on the Exponentially Modified Protein Abundance Index (emPAI) score^12^. Four of the five most enriched binding partners to the more unfolded biosensor (with a *p* < 0.01, Student's *t*-test) were HSP70 family members (3.9-fold more HSPA1B, 1.7-fold more HSPA8) and HSP90 family members (1.9-fold more HS90AB1 and HS90AA1) (Supplementary Table 2). Furthermore, these four chaperones were the most abundant proteins in the immunoprecipitants based on the emPAI score^12^. Hence, we concluded that the basic barnase module is a suitable probe for some of the major chaperones of the QC network.

### Two quantitative measures of QC engagement

**Fig. 3:**
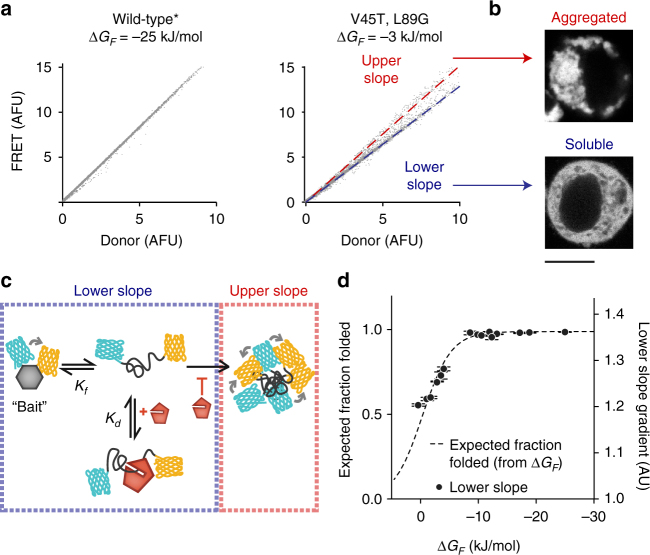
Barnase foldedness and aggregation can be readily assessed by flow cytometry. **a** Flow cytometry analysis to determine FRET. Data points indicate fluorescence signatures of individual cells for donor and FRET channels. **b** Representative confocal images of cells recovered by cell sorting (scale bar=10 µm; “Soluble” image scaled at 4×brightness vs. “Aggregated”). **c** Conceptual framework for how flow cytometry data reports on foldedness vs. aggregation **d** The Lower-slope gradients of barnase mutants measured by flow cytometry (right axis) follows the expected relationship between stability (Δ*G*_*F*_) and fraction folded (left axis, scaled to fit). Each data point reflects one barnase mutant (mean ± SD; three replicates). Note that the AFU or AU scales, while arbitrary, cannot be compared between different panels and figures due to changes in instrument calibration settings

To calculate the extent of QC engagement (which we hereon refer to in terms of holdase activity) we needed to devise a strategy to distinguish the conformers of barnase as depicted in Fig. 1a in intact live cells. To do this, we used flow cytometry to measure donor and FRET (i.e., sensitized emission) channels. For the wild-type* barnase biosensor, we saw a strict linear relationship between donor and FRET emission (Fig. 3a). Because the slope of this dependence is proportional to FRET efficiency (i.e., a higher FRET will result in a greater slope), its linearity indicated that all cells belong to a single FRET population with similar fraction of folded barnase, which we anticipated would be close to 100% based on the Δ*G*_*F*_ value of –25 kJ/mol. By contrast, cells expressing destabilized barnase mutants, such as the V45T, L89G double mutant, segregated into two populations with different FRET slopes (Fig. 3a). Sorted cells from the Lower-slope population were enriched with unaggregated barnase, whereas those in the Upper-slope were enriched with visibly aggregated barnase, accounting for the increased FRET (Fig. 3b). These data suggested that the gradient of the Lower-slope population provided a measure of the balance of folded and unfolded barnase states in the absence of aggregation (Fig. 3c). This conclusion was supported by the Lower-slope gradients of each mutant correlating tightly with the fraction folded expected from the measured *ΔG*_*F*_ (Fig. 3d—the slopes for select data in the figures are presented in Supplementary Data 1; Supplementary Note 1 for more discussion on this point).

**Fig. 4:**
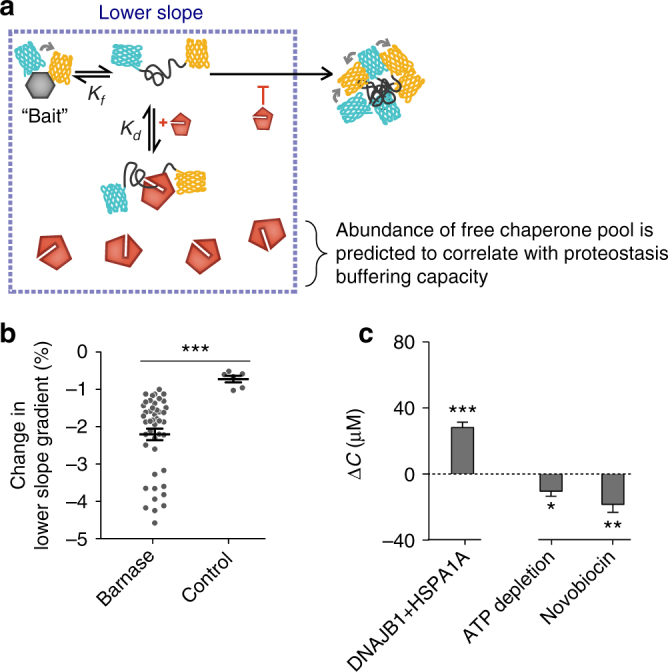
Quantifying proteostasis by changes in latent chaperone concentration available for holdase activity (Δ*C*). **a** Conceptual framework for how chaperone levels affect barnase foldedness. **b** Changes in Lower-slope gradients of the barnase mutants (data points show individual replicates of each mutant) vs. a FRET-positive negative control (mTFP1 cp175-Venus cp173 fusion lacking the barnase kernel; data points show individual replicates) when co-expressed with HSP40 and HSP70 chaperones (DNAJB1 and HSPA1A respectively) relative to baseline conditions (Y66L EGFP co-expression). Means ± SEM shown. **c** Effect of toggling proteostasis on changes in available chaperone capacity (Δ*C*). Plots show means ± SEM of the 12 barnase mutants when coexpressed with DNAJB1 and HSPA1A (compared to Y66L EGFP control; left panel), or treated with proteostasis-modulating drugs (compared to untreated control). Wilcoxon signed rank tests results coded as ***p < 0.001, **p < 0.01, *p < 0.05

To mechanistically explain QC engagement from these data, we considered a simple model for the cell population lacking aggregates (Lower-slope population). In these cells our model assumed three barnase states dominate the molecular pool: folded, free unfolded and unfolded bound to chaperones (and other QC proteins hereon referred to as chaperones for simplicity)^13^. We assumed that the free unfolded state and unfolded-chaperone bound states have similar low FRET values on the basis that HSP70 family proteins (as well as other chaperones) can act as a holdase to unfolded client^14^. We postulated that when the cell has a higher “holdase” capacity there will be a greater pool of latent chaperone (*C*), available to bind unfolded barnase and increasingly partition unfolded barnase from the thermodynamic equilibrium of folding (Fig. 4a). Barnase folding and unfolding rates are typically on the scale of milliseconds to seconds^15^. Assuming that synthesis, degradation and aggregation rates are slower than this—synthesis occurs in mammals on the scale of at least 10 s of seconds per protein^16^ and degradation occurs on the rate of minutes to hours^17^—the ratio between native and free unfolded barnase (*K*_*f*_) will remain the same at equilibrium, but both states will decrease in concentration at higher proteostasis capacity.

To test this model, we overexpressed human chaperones HSPA1A and DNAJB1 (family members of HSP70 and HSP40 respectively). Immunoprecipitation experiments of a biosensor variant tuned to be substantially unfolded (I25A, I96G) verified this treatment produced a greater interaction of HSPA1A with the biosensor as anticipated from elevated holdase activity (Supplementary Fig. 3a–c). And in accordance with the model, this treatment also decreased the Lower-slope values compared to a negative control biosensor lacking the barnase kernel, consistent with a greater holdase activity to the unfolded biosensor (Fig. 4b).

Next we sought to use the model to define the extent of the pool of QC resources capable of holdase activity, defined here as the latent chaperone concentration (*C*). Changes in *C* between a control condition and treatment (Δ*C*) can be determined with knowledge of a binding affinity constant for the average interactions of all chaperones in the cell with barnase (*K*_*d*_), the fraction folded of a given barnase mutant under control (*f*_*c*_) and treatment conditions (*f*_*t*_), folding equilibrium constant *K*_*f*_, and the barnase concentration (*B*)

1 $$\Delta C = \frac{{ - K_dK_f\left( {f_t - f_c} \right)}}{{f_tf_c}} - B\left( {f_t - f_c} \right)\left( {1 + \frac{1}{{K_f}}} \right)$$

Analysis of the Lower-slope data for the set of barnase mutants upon the DNAJB1 and HSPA1A over-expression enabled a calculation of Δ*C* (Fig. 4c) and hence provided a useful scalar measure of QC holdase activity. Details of the analysis are explained in Supplementary Note 2 (for derivation) and Supplementary Note 3 (for application of Equation 1 to our data).

**Fig. 5:**
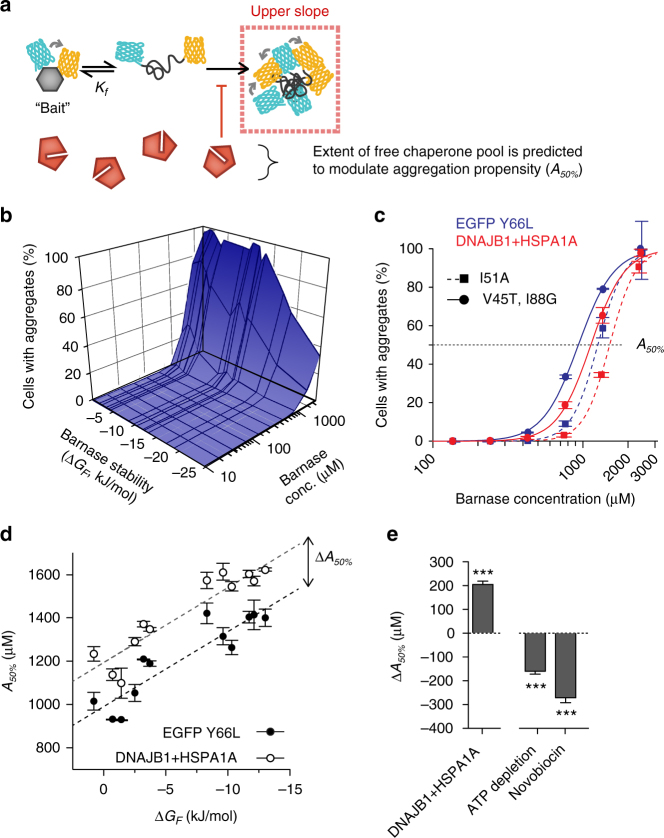
Quantifying proteostasis by changes in biosensor aggregation propensity (*A*_*50%*_). **a** Conceptual framework for how chaperone levels affect barnase-biosensor aggregation. **b** Baseline barnase aggregation “landscape” in cells as measured by the proportion of cells in the Upper-slope flow cytometry population. Data show mean of three replicates for each barnase mutant coexpressed with Y66L EGFP control. **c** Impact on chaperone overexpression on aggregation landscape. Data shows two of the barnase mutants and corresponding treatment regimes. Data points are means ± SD of three replicates. Chaperone treatment vs. control, *p* < 0.0001, extra sum-of-squares F test. **d** Impact of chaperone overexpression on *A*_*50%*_ values. Each data point reflects one mutant (means ± SEM of three replicates). Lines show linear regressions with same slope (preferred model by extra sum of squares F-test, *p* = 0.57). Δ*A*_*50%*_ is the translational offset, calculated for each mutant (example indicated by arrow). **e** Impact of proteostasis on changes in *A*_*50%*_ (Δ*A*_*50%*_). Data show chaperone co-expression (compared to Y66L EGFP negative control), treatment with proteostasis-modulating drugs (compared to untreated control). Data are means ± SEM of 12 mutants of barnase. Results of Wilcoxon signed rank test are shown: Results coded as ***p < 0.001

To provide a quantitative measure of aggregation, we investigated the Upper-slope FRET population and the proportion of cells that fell into this population (Fig. 5a). As anticipated, the proportion of cells containing barnase aggregates increased in correlation with biosensors tuned to more positive Δ*G*_*F*_ values and also to higher protein levels in each cell (Fig. 5b). Overexpression of DNAJB1 and HSPA1A increased the concentration-threshold of aggregation for two of the barnase variants (I51A and V54T, I88G), consistent with chaperone-mediated suppression of aggregation (Fig. 5c). This data indicated that changes in aggregation propensity could be measured by the barnase concentration at which 50% of the cells contained aggregates at a particular time point of expression (*A*_*50%*_) (Fig. 5c). When all the mutants were considered, it was apparent that the *A*_*50%*_ correlated linearly to Δ*G*_*F*_ (Fig. 5d). The DNAJB1 and HSPA1A overexpression treatment offset *A*_*50%*_ by a comparable amount for all barnase mutants (Fig. 5d). These results collectively indicated that the change in proteostasis efficiency can be measured as a single scalar parameter by the translational offset due to the treatment of interest (Δ*A*_*50%*_) (Fig. 5e; further discussion of the *A*_*50%*_ analysis is provided in Supplementary Note 1).

### Using the biosensor to probe how QC manages proteostasis

**Fig. 6:**
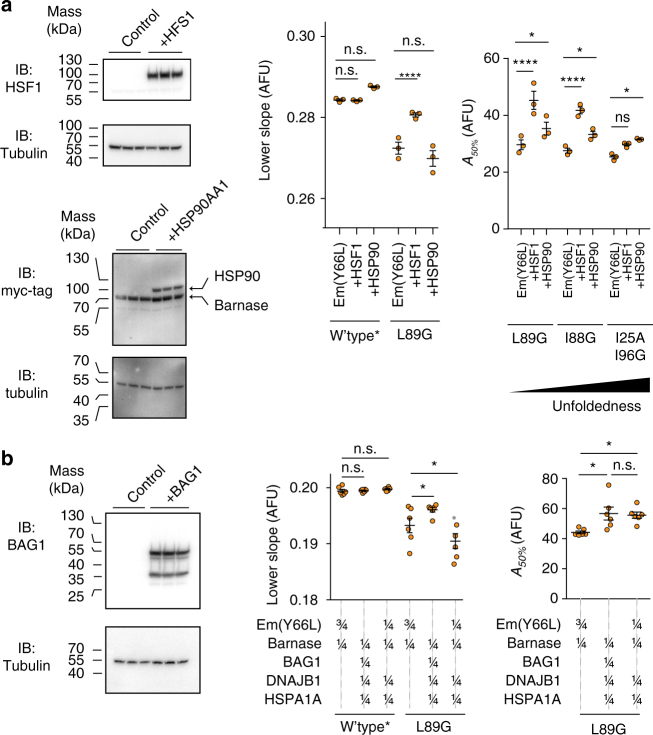
Probing changes in proteostasis through selective activation of different chaperone systems. **a** Investigation of the effect of induction of the heat shock response by HSF1 overexpression or selective overexpression of HSP90. Shown on left are western blots of the HEK293T cells matched for total protein via a BCA assay. These cells were co-transfected with HSF1 (or myc-tagged HSP90 or control of (non-fluorescent) Y66L Emerald fluorescent protein) and the L89G barnase biosensor (which also contains a myc tag). The right graphs show the Lower-slope and *A*_*50%*_ analyses of these treatments and one or three of the biosensor variants as indicated vs. the wild-type* variant. Bars indicate means ± SEM. The right panels were analysed via a two-way ANOVA subjected to a Dunnett’s post-hoc test, results coded as ****p < 0.0001, ***p < 0.001, **p < 0.01, *p < 0.05, ns = > 0.05. **b** Same paradigm as panel a. In this case, BAG1 was cotransfected with the barnase biosensor, HSP40 (DNAJB1) and HSP70 (HSPA1A). The right panels show the proportional dosage of each construct (by mass of DNA) in the transfection. Bars indicate means ± SEM. The right panel Lower-slope graph was analysed via a two-way ANOVA subjected to Dunnett’s post hoc test and the *A*_*50%*_ graph was analysed via a one-way ANOVA subjected to a Tukey’s post-hoc test. Results coded as ****p < 0.0001, ***p < 0.001, **p < 0.01, *p < 0.05, ns = > 0.05

Next we assessed whether we could use the biosensor to measure changes in QC engagement upon stress of proteostasis. First we inhibited HSP90 with novobiocin, which impairs HSP90 activity without activating the heat shock response^18,19^. We predicted this treatment would lead to negative Δ*C* and Δ*A*_*50%*_ values, which was the case (Figs 4d and 5e). Furthermore, the activity of novobiocin could be extracted with this method in terms of a dose response curve, providing an IC_50_ in reasonable accordance with its known value (1.8 mM (our data) c.f. ≈ 700 µM (literature)^18^; Supplementary Fig. 4). As a control, we overexpressed HSP90 family member HSP90AA1, which led to the anticipated reverse response with respect to suppression of aggregation although the change in holdase activity was not significant (Fig. 6a). As a second test for stress, we depleted ATP levels using a glycolysis inhibitor (2-deoxy-d-glucose) in combination with an inhibitor of oxidative phosphorylation (valinomycin)^20,21^ (Supplementary Fig. 5). Under such conditions, we predicted that chaperone networks would be impaired from functioning, and hence lead to a net loss of engagement with client. This appeared to be the case, with a negative Δ*C* (Fig. 4d) resulting as well as a negative Δ*A*_*50%*_, (Fig. 5e).

Next we explored the capacity of the biosensor system to gain insight into the mechanics of QC systems that regulate proteostasis. First, we applied a selective inhibitor of HSP70 ATPase activity VER-155008, which binds competitively to the ATP binding pocket of HSP70 family proteins^22^. While this inhibitor does not prevent client binding in vitro^23^, it did lead to an increase in the Lower-slope values and decrease in *A*_*50%*_ consistent with an overall reduction in the pool of chaperone supply with dynamic capacity to act as a holdase and suppress aggregation (Supplementary Fig. 6).

Next we probed HSP70-HSP40 holdase activity in greater context of nucleotide exchange factors (NEFs), which catalyze the disengagement of client bound to HSP40-HSP70 to complete the chaperone cycle^24–26^. As described above (Fig. 4c, d), overexpression of HSPA1 and DNAJB1 increased the net unfolded pool of the biosensor through a gain in holdase activity. This suggested that overloading a cell with HSPA1 and DNAJB1 by their selective overexpression created a bottleneck in QC capacity to complete the chaperone cycle. To test this possibility, we co-expressed BAG1, the NEF co-factor to HSPA1, with HSPA1 and DNAJB1 (Fig. 6b). This treatment significantly increased foldedness of a biosensor tuned to be moderately unfolded (L89G) compared to when HSPA1 and DNAJB1 were overexpressed without BAG1. This is hence consistent with a decrease in HSP70 holdase activity as anticipated by a greater NEF activity to facilitate release of client bound to HSPA1 and DNAJB1. Assessment of aggregation by Δ*A*_*50%*_ analysis revealed the NEF to not provide any further enhancement to proteostasis than HSPA1 and DNAJB1 overexpression without BAG1, suggesting that the holdase activity may be sufficiently effective to mitigate inappropriate aggregation (Fig. 6b).

An interesting result from this experiment was that the NEF-HSP40-HSP70 overexpression increased foldedness of the biosensor to a greater extent than what was observed under baseline conditions (Fig. 6b). This suggested that the HSP70 chaperone cycle did not operate at maximum potential under normal conditions whereby NEF activity was the rate limiting step. To further probe this possibility, we overexpressed HSF1, which induces expression of the heat shock response genes (Fig. 6a). This treatment significantly increased foldedness of the L89G variant biosensor (and also suppressed its aggregation) beyond baseline conditions (Fig. 6a). Hence, this supported the conclusion that HSP70 chaperone activity is rate-limited under normal conditions by NEF activity and that this can be overcome by a coordinated upswing in cellular QC resources driven through the heat shock response gene elements.

## Discussion

We describe here a strategy for quantifying the holdase activity of the QC network as an indicator of proteostasis health. Using barnase as a bait protein to the QC we were able to define the extent to which the QC system altered the folding equilibrium and aggregation and also validate how manipulation of QC machinery alters the biosensor readout in a predictable way. The key objective of the biosensor was to provide a simple numerical metric of “holdase” activity and capacity to suppress aggregation. As such, this system provides a quantitative measure of the health of proteostasis. However, as indicated by the studies with selective chaperone overexpression and proteomics, the biosensor also has capacity for nuanced analysis of the specific changes in chaperones that interact with the biosensor under different conditions. One area of potential application is in the study of selectivity in how different HSP70 proteins respond to client under different stresses. Prior work has suggested HSP70 family members display selectivity in function: some are better at refolding misfolded proteins and others at preventing aggregation^27^. Others seem to be involved in protein disaggregation^28^. The biosensor platform described here provides a manner to explore the dual role of holdase activity and aggregation of individual HSP70 proteins (e.g., by their selective knock out) in an intact proteostasis system. An interesting finding was how the HSP70 chaperone cycle appeared to not be operating a maximal capacity under baseline conditions. This may provide a mechanism for HSP70 to accumulate client as part of HSP70-mediated client triage to other nodes of the QC system, such as degradation^29^. It may also provide QC systems an instant buffering strategy after an acute stress during the time period for stress responses to synthesize additional QC resources.

Collectively, the new biosensor system and methods described here offer promise to probe mechanisms intersecting proteostasis and disease. In particular the biosensor system has great potential to be developed into a tool to measure early changes in neurodegenerative disease where early diagnostics are desperately needed. The development of treatments for neurodegenerative diseases including Huntington’s (HD), Alzheimer’s (AD) and Motor Neuron Disease (MND) remains one of the toughest scientific challenges of our times. Between 2002 and 2012 only one of 244 AD clinical trials yielded a treatment with therapeutic benefit^30^. Similarly Motor Neuron disease remains largely untreatable with only two drugs approved by the US Food and Drugs Administration that mildly delay disease progression (edaravone and riluzole)^31,32^. The failure of clinical trials in neurodegenerative diseases may arise from trials beginning too late in the disease course. In turn, beginning trials at presymptomatic stages of disease has been hampered by a lack of biomarkers of presymptomatic disease progression^33^. Hence approaches, such as ours described here, provide important foundation stones for tackling this great challenge.

## Methods

### Expression constructs

A toolkit for FRET biosensor comprising a combinatorial library of circularly permuted mTFP1 and Venus fluorescent proteins (cpFRET library in pTriEx4 expression vector) was used^34^. Barnase mutants were synthesized (Thermo Fisher Scientific) and inserted via PCR-mediated cloning with XmaI and NotI flanking restriction sites between BspEI (complementary to XmaI) and NotI sites of the cpFRET library such that barnase was fused to mTFP1 and Venus at the amino and carboxy termini, respectively. Additional single mutations were isolated from double-mutant constructs using BspEI and XbaI restriction sites. All constructs were verified by sequencing and the basic construct sequence is shown in Supplementary Table 3. Plasmids expressing HSPA1A, DNAJB1 and mCherry were prepared as described previously^35,36^. pCMV6-AC BAG1 (cat# SC319483), pCMV6-AC HSF1 (cat# SC321225) and pCMV6-Entry myc-HSP70AA1 (cat# RC212496) were purchased from Origene.

### Cell culture

AD293 and HEK293 cell-lines (from lab cultures orginally obtained from ATCC) were used in this study and tested and cleared for mycoplasma. Cells were not tested for cross-contamination of other cell lines or misidentification. AD293 cells were maintained in Dulbecco’s modified Eagles Medium (DMEM) supplemented with 2 mM l-glutamine, 200 U mL^−1^ penicillin/streptomycin and 10% v/v fetal bovine serum (Thermo Fisher Scientific) in a humidified 37 °C incubator with 5% v/v atmospheric CO_2_. HEK293T cell-lines were maintained as for AD293 cells except DMEM was supplemented with 2 mM L-alanyl-L-glutamine, 100 U mL^–1^ penicillin/streptomycin and 10% v/v fetal bovine serum. For biosensor screening, 2 × 10^5^ AD293 cells were seeded on poly-L-lysine coated 12-well plates and transfected using 1.6 µg DNA and 4 µL Lipofectamine 2000 as per the manufacturer’s directions. For urea denaturation, flow cytometry and microscopy, 5 × 10^4^ HEK293T cells were seeded on poly-l-lysine coated 48-well plates (Corning) or 8-well μ-slides (for microscopy, Ibidi). Cells were transfected using 0.25 μg DNA, 0.5 µL P3000 reagent and 0.75 µL Lipofectamine 3000 according to the manufacturer’s instructions.

### Drug treatments

To deplete ATP, cells were kept in glucose-free DMEM (Thermo Fisher Scientific) for 16 h then treated with 10 mM 2-deoxy-d-glucose (Sigma) and 0.5 µM valinomycin (Sigma) in glucose-free DMEM for 45 min. ATP levels in ATP-depleted and control (untreated) cells were measured using the ATPlite luminescence assay system (Perkin Elmer) on a ClarioStar microplate reader, as per the manufacturer’s protocol. HSP90 was inhibited with 800 µM novobiocin in maintenance media for 6 h. HSP70 was inhibited with 20 µM VER-155008 (Sigma cat #SML0271) in maintenance media for 18 h.

### FRET biosensor screen

The cpFRET library containing wild type* (H102A) barnase was transfected into AD293 cells. 24 h post-transfection cells were washed with PBS then lysed by extrusion through a 27 gage syringe in native lysis buffer (20 mM Tris pH 8.0, 2 mM MgCl_2_, 1% v/v Triton X-100, 1 × EDTA-free protease inhibitor (Roche), 150 mM NaCl, 20 U mL^–1^ benzonase, 1 mM PMSF). Lysate was centrifuged at 100,000 × g for 30 min at 4 °C to remove cell debris. 80 µL supernatant was added to 200 µL native lysis buffer or native lysis buffer with 6 M urea in a 96-well plate. mTFP1 was excited at 462 nm and emission spectra were collected from 480 to 600 nm using a Varioskan Flash microplate reader (Thermo Fisher Scientific). Relative FRET efficiency was calculated as *A*/(*D*+*A*); that is, fluorescence intensity at the acceptor (*A*) maximum (532 nm) divided by the sum of intensities at the donor (*D*) (492 nm) and acceptor maxima. Because the readings were ratiometric, we deemed it unnecessary to normalize protein concentration in the lysates.

### Recombinant production of the Venus cp173 construct

The Venus cp173 construct in the pTriEx based vectors was expressed in T7 Express *E. coli* (NEB) using ampicillin as the selection antibiotic. 10 mL of an overnight starter culture (grown in 2 × YT at 37 °C in a shaking incubator), was inoculated in 1 L of 2 × YT and grown to an OD_600nm_ of 0.5 AU at 37 °C in a shaking incubator. The culture was cooled to 18 °C and then expressed was induced with 0.4 mM Isopropyl β-D-1-thiogalactopyranoside with cells grown overnight at 18 °C in a shaking incubator. Cells were pelleted (5000 × *g*; 4 °C) and resuspended in 100 mM Tris, pH 8.0 supplemented with Complete EDTA-free protease inhibitor cocktail (Roche) and 1 mM phenylmethylsulfonyl fluoride. Hen egg white lysozyme was added to a concentration of 1 mg mL^–1^ and the lysate was frozen at –20 °C. The lysate was thawed, Benzonase nuclease was added according to the manufacturer’s instructions (EMD-Millipore). The debris was pelleted and removed by centrifugation (16,000 × *g*; 20 min; 4 °C). Imidazole was added to the supernatant to a concentration of 5 mM and the solution was applied to a 1 mL His-tag column pre-equilibrated in binding buffer (PBS and 5 mM imidazole). The column was washed with 20 mL column buffer and then with PBS and 25 mM imidazole until no proteins further eluted (as assessed by Bradford assay). The column was then washed with 10 mL PBS and 50 mM imidazole before elution with PBS and 200 mM imidazole. Eluted proteins from the most concentrated fractions were buffer exchanged into PBS using a PD-10 column (GE Healthcare). Proteins were immediately snap frozen in liquid nitrogen and stored at −80 °C until further use.

### Recombinant production of the barnase biosensors

The barnase constructs in the pTriEx based vectors were expressed in T7 Express *E. coli* (NEB) using ampicillin as the selection antibiotic. 10 mL of an overnight starter culture (grown in 2 × YT at 37 °C in a shaking incubator), was inoculated in 100 mL of 2 × YT and grown to an OD_600 nm_ of 0.5 AU at 37 °C in a shaking incubator. The culture was cooled to 18 °C and expression was induced with 0.4 mM Isopropyl β-D-1-thiogalactopyranoside with cells grown overnight at 18 °C in a shaking incubator. Cells were pelleted (5000 × *g*; 4 °C) and resuspended in phosphate buffered saline (PBS) supplemented with Complete EDTA-free protease inhibitor cocktail (Roche). Hen egg white lysozyme was added to a concentration of 1 mg mL^−1^ and the lysate was frozen at −20 °C. The lysate was thawed, Benzonase nuclease was added according to the manufacturer’s instructions (EMD-Millipore). The debris was pelleted and removed by centrifugation (16,000 × *g*; 15 min; 4 °C). Imidazole was added to the supernatant to a concentration of 20 mM and the solution was applied to a His SpinTrap column (GE Life Sciences) pre-equilibrated in binding buffer (PBS and 20 mM imidazole). Proteins were purified as per the manufacturer’s directions, using PBS and 200 mM imidazole as the elution buffer. Proteins were used immediately.

### Urea denaturation stability measurements

384-well plates were prepared with 80 µL of a concentration series of urea (0 M to ~ 6 M) in phosphate buffered saline (PBS). For the recombinant purified proteins, 5 µL of protein was used in the assay. For the curves performed with cell lysates, cells were lysed 24 h after transfection by pipetting in native lysis buffer. Aggregates and cell debris were pelleted by centrifugation at 16,000 × *g* for 10 min at 4 °C. 5 µL supernatant was added to each urea concentration. Samples were not matched for protein concentration; however, as the measurements were ratiometric and both fluorophores were on the same molecule, concentration was not expected to influence results. Fluorescence readings (430 nm excitation, 492 nm emission and 532 emission) were measured at 23 °C using a ClarioStar microplate reader every 15 min for 4 h. Readings were stable for the duration of the experiment. Relative FRET efficiencies (calculated as *A*/(*D*+*A*) as per the FRET biosensor screen) were averaged across readings and fit to a two-state unfolding model with terms for pre- and post-transition baselines (Equation 2).

2 $${\mathrm{FRET}} = \frac{{\left( {\alpha _N + \beta _N \cdot \left[ U \right]} \right) + \left( {\alpha _D + \beta _D \cdot \left[ U \right]} \right) \cdot {\mathrm{e}}^{\frac{{ - m}}{{RT}}\left( {D_{\it{50\% }} - [U]} \right)}}}{{1 + {\mathrm{e}}^{\frac{{ - m}}{{RT}}\left( {D_{\it 50\% } - [U]} \right)}}}$$

Where [*U*] is the urea concentration, *D*_*50%*_ is the urea concentration at which barnase is 50% denatured, *m* is the cooperativity value and *m*(*D*_*50%*_−[*U*]) = Δ*G*_*F*_, and *α* and *β* describe the FRET baselines of barnase in the native (*α*_*N*_, *β*_*N*_) and denatured (*α*_*D*_, *β*_*D*_) conformations. That is, *α* is the FRET signal of barnase in the native (*α*_*N*_) or denatured (*α*_*D*_) conformation when [*U*] = 0 and *β* is the rate of change of the FRET signal with increasing [*U*]. As small error in the cooperativity value *m* has large impact on Δ*G*_*F*_, *m* was constrained as a shared parameter for all mutants. To enable fits for destabilized mutants that are partially unfolded at 0 M urea, pre- and post-transition baselines were shared for all mutants. Reported Δ*G*_*F*_ were averages of three experiments fit independently.

### Microscopy

To estimate cell volume, cells expressing Venus (cp173) were imaged 24 h after transfection on a Leica TCS SP5 Confocal microscope with a HCX APO CS 63 × 1.40 Oil objective and 1 Airy pinhole as Z-stacks in 0.21 µm steps (514 nm excitation, 520–650 nm emission) and analyzed using ImageJ. To measure FRET in intact cells, cells were imaged with a Leica SP5 confocal microscope using a HCX APO CS 63 × 1.40 Oil objective and 1 Airy pinhole. Emission spectral scans were acquired with a 405 nm excitation laser, from 480 to 576.6 nm in 3.45 nm steps with a collection bandwidth window of 10 nm.

### Immunoprecipitation for western blotting

In total 1.25 × 10^5^ HEK293T cells were seeded into 25 cm^2^ flasks and transfected the following day with 6.25 µg DNA constructs, 12.5 µL P3000 and 18.75 µL Lipofectamine 3000 (Life Technologies) according to the manufacturer’s instructions (Life Technologies). Cells were harvested at 24 h post-transfection for drug treatments and 48 h for co-expressions. Cells were harvested by gently pipetting with PBS. Cells were pelleted by centrifugation at 120 × *g* for 6 min and resuspended in lysis buffer (0.5% v/v IGEPAL, 50 mM Tris, 5 mM MgCl_2_, pH 7.4 supplemented with EDTA-free protease inhibitor (Roche). Lysate was incubated on ice for 10 min. Debris was removed by centrifugation (13,000 rpm; 10 min; 4 °C) and the supernatant placed in a new microcentrifuge tube. For samples matched for barnase fluorescence, fluorescence was measured on a ClarioStar microplate reader (ex = 513 ± 10 nm, em = 530 ± 30 nm). 20 µL of GFP-Trap_MA (Chromotek) beads prewashed in wash buffer (0.5% v/v IGEPAL, 50 mM Tris, 150 mM NaCl, 5 mM EDTA, pH 7.4) was added to each sample and the mixture was incubated for 2 h at 4 °C with constant rotation. The unbound fraction was removed from beads immobilized with a magnet, and beads were washed three times with wash buffer at room temperature. Proteins were eluted off the beads by boiling the beads in SDS-PAGE Laemmli sample buffer for 10 min.

### Western blotting

Proteins were transferred to PVDF membrane, blocked in blocking buffer (5% w/v skim milk powder in PBS-T) for 1 h at room temperature and then incubated with primary antibody in blocking buffer. The following antibodies were incubated overnight at 4 °C: anti-HSPA1A (Origene, cat #TA500772, 1:10,000), anti-HSF1 (Abcam, cat #ab52757, 1:40,000), anti-BAG1 (Abcam, cat #ab32109, 1:750), and anti-myc (Thermofisher, cat #13–2500, 1:1,000). The blots were washed in PBS-T and then incubated with either anti-rabbit secondary antibody (Invitrogen, cat #65-6120, 1:20,000) or anti-mouse secondary antibody (Invitrogen, cat #31430, 1:20,000) in PBS-T for 1 h at room temperature. Proteins were detected by an enhanced chemiluminescence kit (Clarity, BioRad). The uncropped blots are shown in Supplementary Fig. 7.

### Sample preparation for proteomics

In total 3.5 × 10^6^ HEK293T cells were seeded into 75 cm^2^ flasks and transfected the following day with either WT* or I25A, I96G barnase constructs (18.75 µg DNA, 37.5 µL P3000 and 56.25 µL Lipofectamine 3000 (Life Technologies)) according to the manufacturer’s instructions (Life Technologies). The experiment was designed as 4 biological matched pair replicates. Media was refreshed 5 h after transfection. At 24 h post-transfection, cells were gently rinsed with PBS and harvested in PBS by gently pipetting. Cells were pelleted (120 × *g*; 6 min; room temperature) and resuspended in 1 mL PBS and pelleted again (4000 × *g*; 6 min; room temperature). The pellet was resuspended in ice-cold 200 µL HENG buffer (50 mM HEPES-KOH, pH 7.9, 150 mM NaCl, 20 mM Na_2_MoO_4_, 2 mM EDTA, 5% v/v glycerol, 1 mM PMSF and Complete EDTA-free protease inhibitor (Roche)). Cells were mechanically lysed using a cryomill (Precellys 24; Bertin) after addition of 40 µL 0.15 mm zirconium oxide beads using 3 × (30 s on, 30 s off) cycles at 6800 rpm with temperature monitored and maintained at 10 °C or less. The resultant lysate was supplemented to 500 µL with HENG buffer and pelleted (16,000 × *g*; 10 min; 4 °C). The supernatant was removed and matched for Venus fluorescence (ex = 514 nm, em = 527 nm) using a platereader (ClarioSTAR; BMG). 350 µL of lysate was added to 30 µL GFP-Trap agarose beads (Chromatek) pre-washed and equilibrated in HENG buffer. The solution was incubated for 2 h at 4 °C with constant rotation. Beads were collected by pelleting (2000 × *g*; 2 min; 4 °C) and washed twice with HENG buffer by pelleting and resuspension. Beads were then washed twice more with 1 mM triethylammonium bicarbonate (TEAB) buffer. Proteins were eluted by addition of 30 µL 0.1% v/v formic acid, 5% v/v trifluoroethanol, 1 mM tris(2-carboxyethyl)phosphine for 5 min at room temperature. The supernatant was collected after pelleting (2000 g; 2 min; room temperature) and adjusted to a final concentration of 100 mM TEAB by addition of 1 M stock solution (and the pH was validated to be about 7 after this treatment). Proteins were reduced using 10 mM tris (2-carboxyethyl)phosphine, pH 8.0, and alkylated with 10 mM iodoacetamide for 45 min and then digested by addition of 0.25 µg trypsin and incubation overnight at 37 °C. Peptides (in a volume of 50 µL) were differentially labelled by reductive dimethyl labelling using 2 µL of 4% (vol/vol) formaldehyde –CH_2_O (light label), CD_2_O (medium label), ^13^CD_2_O (heavy) (mixed design across replicates) and 2 µL of 0.6 M sodium cyanoborohydride for 1 h at room temperature. The reaction was quenched by addition of 8 µL of 1% ammonium hydroxide followed by 8 µL of neat formaldehyde.

### NanoESI–LC–MS/MS analysis

Samples were analysed by nanoESI–LC–MS/MS using a Q Exactive Plus mass spectrometer (Thermo Scientific, San Jose, CA) fitted with a nanoflow reversed-phase-HPLC (Ultimate 3000 RSLC, Dionex). The nano-LC system was equipped with an Acclaim Pepmap nano-trap column (Dionex−C18, 100 Å, 75 μm × 2 cm) and an Acclaim Pepmap RSLC analytical column (Dionex−C18, 100 Å, 75 μm × 50 cm). Typically for each LC-MS/MS experiment, 5 μL of the peptide mix was loaded onto the enrichment (trap) column at an isocratic flow of 5 μL min^−1^ of 3% CH_3_CN containing 0.1% formic acid for 5 min before the enrichment column was switched in-line with the analytical column. The eluents used for the LC were 0.1% v/v formic acid (solvent A) and 100% CH_3_CN/0.1% formic acid v/v (solvent B). The gradient used (300 nL min^−1^) was from 3 B to 20% B for 35 min, 20 B to 45% B in 8 min, 45 B to 80% B in 2 min and maintained at 80% B for the final 3 min before equilibration for 6 min at 3% B prior to the next analysis. All spectra were acquired in positive mode with full scan MS spectra scanning from m/z 375–1400 in the FT mode at 70,000 resolution after accumulating to a target value of 3.00e^6^ with maximum accumulation of 50 ms. Lockmass of 445.120024 was used. Data dependant HCD MS/MS of the 15 most intense peptide ions with charge states >1 was performed, using an isolation width of 1.2, a target value of 1.00e^5^, a maximum accumulation time of 120 ms, a normalized collision energy of 30%, and a 35,000 mass resolving power. Dynamic exclusion was used for 30 s.

Data analysis was carried out using Proteome Discoverer (version 2.1.0.81; Thermo Scientific) with the Mascot search engine (Matrix Science version 2.4.1). Data were filtered against the Swissprot *Homo sapiens* database (version 2015_07: Jun-24, 2015; 548872 entries). The search was conducted with 20 ppm MS tolerance, 0.8 Da MS/MS tolerance, 2 missed cleavages allowed. The following modifications were allowed: Oxidation (M), Acetylation (Protein N-term), Dimethylation (K), Dimethylation (N-Term), ^2^H(4) Dimethylation: (K), ^2^H(4) Dimethylation (N-term), ^2^H(6)^13^C(2) Dimethylation (K), ^2^H(6)^13^C(2) Dimethylation (N-term) (Variable); Carbamidomethyl (C) (Fixed). The false discovery rate (FDR) maximum was set to 0.1% at the peptide identification level (actual was 0.06% for each replicate) and 1% at the protein identification level. Proteins were filtered for those containing at least one unique peptide in all four replicates. The common contaminant, Keratin, was excluded from the dataset. Peptide quantitation was performed in Proteome Discoverer v.2.1.0.81 using the precursor ion quantifier node. Dimethyl labelled peptide pairs (between two comparison of light, medium or heavy) were established with a 2 ppm mass precision, a signal to noise threshold of 3, and filtered for a minimum Mascot ion score of 30. The retention time tolerance of isotope pattern multiplets was set to 0.8 min. Three single peak or missing channels were allowed for peptide identification. The protein abundance in each replicate was calculated by summation of the unique peptide abundances that were used for quantitation (light, medium and-or heavy dimethyl derivatives). In the cases where quan values were missing or were apparent outliers to the other two replicates, raw data was manually checked and added or adjusted where relevant. The protein ratios (from two of the light, medium and heavy labels) were manually calculated from the protein abundances as described previously^37^. Data were normalized to the Venus protein peptide abundances in the datasets. These correction values were multipliers of (replicate 1: 3.583), (replicate 2: 3.45), (replicate 3: 4.284) and (replicate 4: 5.027). Proteins were excluded that had an SD greater than 60%. Student’s t-test was performed to calculate the statistical significance.

### Flow cytometry

After 24 h (drug treatments) or 48 h (cotransfections) of post-transfection, cells were washed once in PBS then harvested by gentle pipetting in PBS and transferred to a 96-well U-bottom microplate. 100 µL cell suspension was analysed at 3 µL s^−1^ using the high throughput sampler in an LSRFortessa flow cytometer equipped with 405, 488, and 561 nm lasers (BD Biosciences). Forward scatter threshold was set to 5000. Acceptor (Venus) fluorescence was collected with the 488 nm laser and FITC (530/30) filter. Acceptor sensitized emission (FRET) and donor (mTFP1) fluorescence were collected with the 405 nm laser with PE (575/25) and V500 (525/50) filters, respectively. All flow cytometry data were processed with FlowJo (Tree Star Inc) to exclude cell debris, cell aggregates and untransfected cells.The Venus channel was compensated to remove bleedthrough from mTFP1 and FRET channels. mTFP1, FRET and Venus data were exported as csv files and analyzed in MATLAB (see section on data analysis). The gating strategy is explained in the associated Protocols Exchange manuscript^38^.

Cells were sorted using a Becton Dickinson FACSAria III sorter at the University of Melbourne Brain Centre flow cytometry facility. FRET and mTFP1 fluorescence were collected with the 405 nm laser with 582/15 and 510/50 filters, respectively. Cells were fixed in 2% v/v paraformaldehyde immediately after sorting and imaged on cover slips using Venus fluorescence as described above (without Z-stacks).

### Data analysis

Flow cytometry csv files were analyzed in MATLAB (MathWorks) to automatically classify cells as diffuse, or aggregated. The script is available upon request. The rational of the analysis is described here. The Lower-slope and Upper-slope populations can be visually distinguished on a plot of the FRET channel against the donor channel. However, since the Lower-slope gradient differed for each barnase mutant, we could not use of a single Upper-slope gate for aggregate classification. Our approach was to fit the slope of the Lower-slope population, then classify any cells with substantially higher FRET than this belonging to the Upper-slope population. Since many datasets contained high numbers of cells with barnase aggregation, fitting methods designed to ignore outliers were insufficient to exclude high-FRET cells from the fit. To overcome this we (i) implemented a pre-processing step in FlowJo to immediately exclude very high-FRET cells (i.e., higher FRET than all wild-type* barnase cells) from the fit, then (ii) performed a robust fit multiple times, excluding data that deviated significantly above the slope after each iteration. To determine significant deviation above the slope, we used the standard deviation of cells expressing wild-type* barnase (which contains no barnase aggregates) from the slope as an estimate of the expected standard deviation of diffuse cells around the slope for all mutants. We excluded cells that were greater than 2 standard deviations above the slope from the subsequent fitting iteration. We found that four iterations was sufficient to fit the Lower-slope population satisfactorily.

For analysis of the Lower-slope population the Venus acceptor fluorescence was restricted to a range up to 0.2 of the maximum dynamic range (which corresponded to typically 560–4600 AFU on the BD LSRFortessa flow cytometer). This concentration range provided a trade-off between having low barnase concentration at which the model predicts chaperone engagement will be most pronounced, having enough cells for high quality data and having sufficient signal above background to accurately determine the Lower-slope gradient.

For analysis of *A*_*50%*_, cells were binned into expression levels using Venus fluorescence, with 9 logarithmic bins evenly spanning the lower and upper bounds of fluorescence (e.g., 10^2.5^, 10^2.75^, 10^3^, 10^3.25^, 10^3.5^, 10^3.75^, 10^4^, 10^4.25^, and 10^4.5^ AFU). The percent of cells with aggregates (i.e., the Upper-slope population) were calculated for each expression bin. For each dataset, a Boltzmann sigmoidal curve was fit to the relationship between percent of cells with aggregates and log_10_(expression bin midpoint) using Graphpad Prism. For the most stable mutants there was very little aggregation, even at high expression level, resulting in large uncertainty in the fits. Therefore, data from wild-type*, V45T, and I55G were excluded from the analysis. The expression level at which there was 50% aggregation (*A*_*50%*_) was determined from the sigmoidal fits. For hypothesis testing (i.e., to determine whether a treatment resulted in a significant change in aggregation propensity) the difference between average *A*_*50%*_ of control and treatment was calculated for each barnase mutant (excluding wild-type*, V45T, and I55G). A Wilcoxon signed rank test was used to calculate whether the difference was significantly different to zero, treating the twelve mutants as replicate values.

Equation 1 was derived as described in Supplementary Note 2 and applied to our data as described in Supplementary Note 3.

### Statistics

All statistics (other than the Proteomics) were performed in Prism software version 5 (GraphPad). The statistical tests and results are described in the figure legends.

### Data availability

Raw data not presented in the manuscript are available upon request.

## Electronic supplementary material

Supplementary Information

Description of Additional Supplementary Files

Supplementary Data 1
