# Supplementary material for: A biosensor-based framework to measure latent proteostasis capacity
Source: Nat Commun. 2018 Jan 18;9:287. doi: 10.1038/s41467-017-02562-5 (PMC5773518; doi:10.1038/s41467-017-02562-5)
Supplement: Supplementary file 1 — Supplementary Information [file 41467_2017_2562_MOESM1_ESM.pdf]

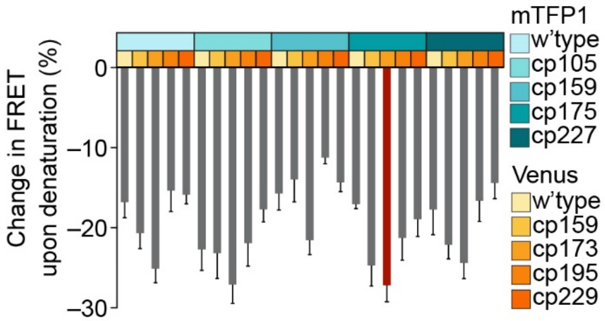


**Supplementary Fig. 1. Screening circularly permuted variants of mTFP1 and Venus for maximum FRET change upon barnase denaturation.** Wild-type* barnase was cloned into a combinatorial library of circularly permuted variants of mTFP1 and Venus at the N- and C-termini, respectively. The relative FRET efficiency of mammalian lysate expressing each construct was measured under native and denaturing (~6 M urea) conditions. FRET change upon denaturation is expressed as a percent of the native FRET signal. Data show mean ± SEM of three replicate experiments. Barnase fused to mTFP1 cp175 and Venus cp173 (used for remaining experiments) is highlighted in red.

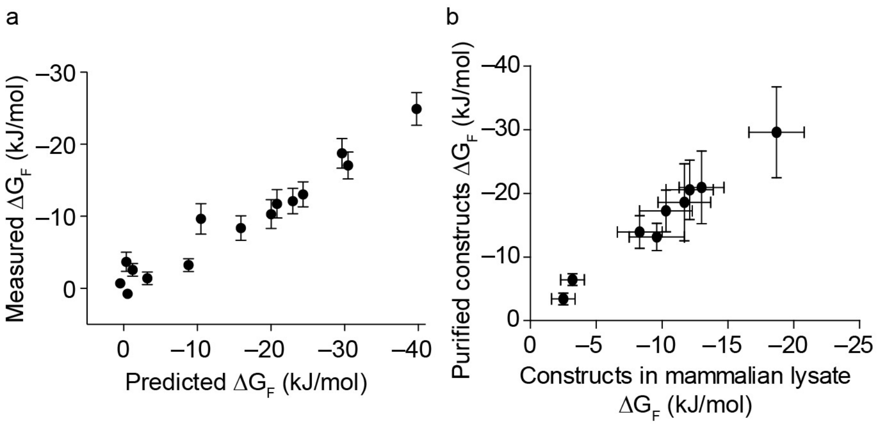


**Supplementary Fig. 2. Stability of the barnase fusions.** **a)** Measured stability of barnase fusions compared to predicted values from literature sources. To combine literature values from multiple sources [1,2], the wild-type* $\Delta G_F$=39.7 kJ/mol was used, and values were calculated from the reported $\Delta\Delta G_F$. For double mutants, $\Delta\Delta G_F$ were assumed to be additive. Literature values were measured by urea or guanidinium chloride denaturation in 50 mM Mes pH 6.3 at 25 °C. Values of barnase fusion constructs were measure by urea denaturation in phosphate buffered saline pH 7.4 at 25 °C using mammalian lysate. Measured and predicted values correlate well (Pearson's correlation coefficient = 0.9716); however measured values are more positive (less stable) than predicted. Data show mean ± SEM of three independent urea denaturation curves. **b**) Comparison of $\Delta G_F$ values from purified proteins versus in mammalian lysate. This time the purified proteins were more stable (by about 1.5-fold) than in lysate. Hence, it appeared that factors in the lysate or buffer conditions led to more positive $\Delta G_F$ values; the lower stability of proteins in cells has been reported for other proteins suggesting this could be a more general property of cellular solutes [3]. Nonetheless, the absolute values are not critical for our analysis – rather they provide a reference point to measure deviations from under a given treatment condition.

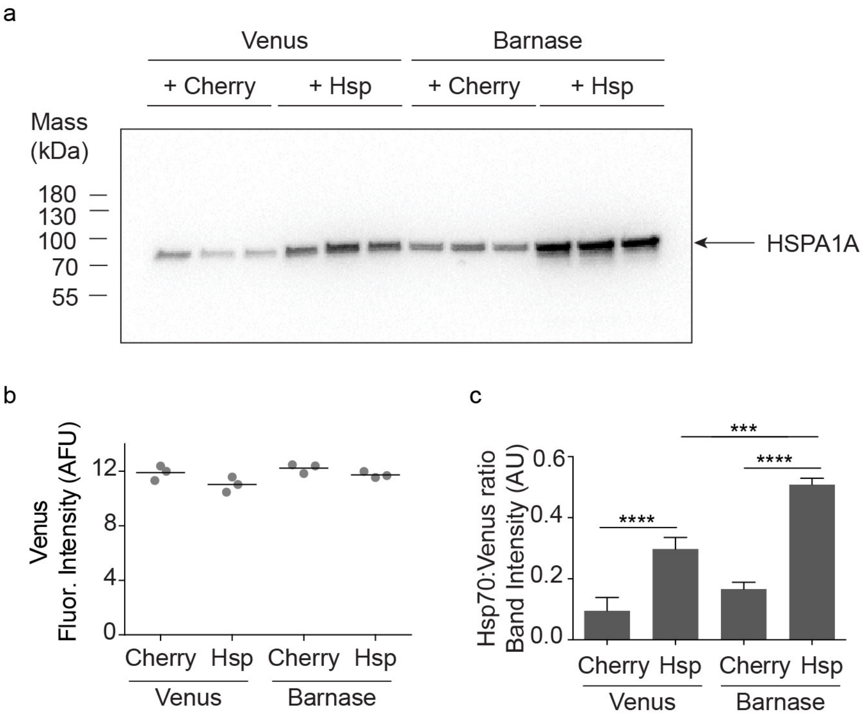


**Supplementary Fig. 3. Overexpression of Hsp40 (DNAJB1) and Hsp70 (HSPA1A) promote greater HSP70 association with barnase. a)** Immunoprecipitants of barnase or a control (Venus fluorescent protein) by GFP-Trap in HEK293T cells. Cells were co-transfected with I25A, I96G version of the biosensor (or Cherry fluorescent protein as a control) and DNAJB1 and HSPA1A. Shown is a Western Blot probed for HSPA1A. **b**) Shown are fluorescence intensity measurements (ex = 513 ± 10 nm, em = 573 ± 60 nm) of input lysate for the immunoprecipitations. Input was matched for equivalent biosensor fluorescence. **c**) Quantitation of data in panels a and b. Data are means ± SD of 3 replicates. Results of ANOVA and Tukey's post hoc test are shown: Results coded as *** $p<0.001$, **** $p<0.0001$.

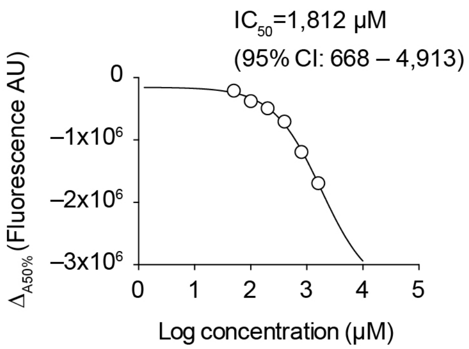


**Supplementary Fig. 4. Application of biosensor to probe proteostasis upon Hsp90 inhibition with novobiocin.** Dose response curve for novobiocin as calculated by the $A_{50\%}$ values. Shown are HEK293T cells expressing the I25A, I96G biosensor. Cells were transfected and after 18 h were treated with novobiocin for a further 6 h prior to analysis.

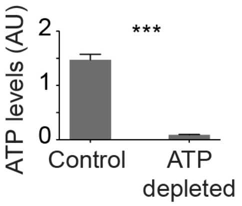


**Supplementary Fig. 5. ATP depletion treatment reduced ATP levels (measured by luminescence assay) to approximately 6% of control values.** Data show mean ± SEM of 6 replicate wells. ***p<0.001, Unpaired t-test with Welch's correction.

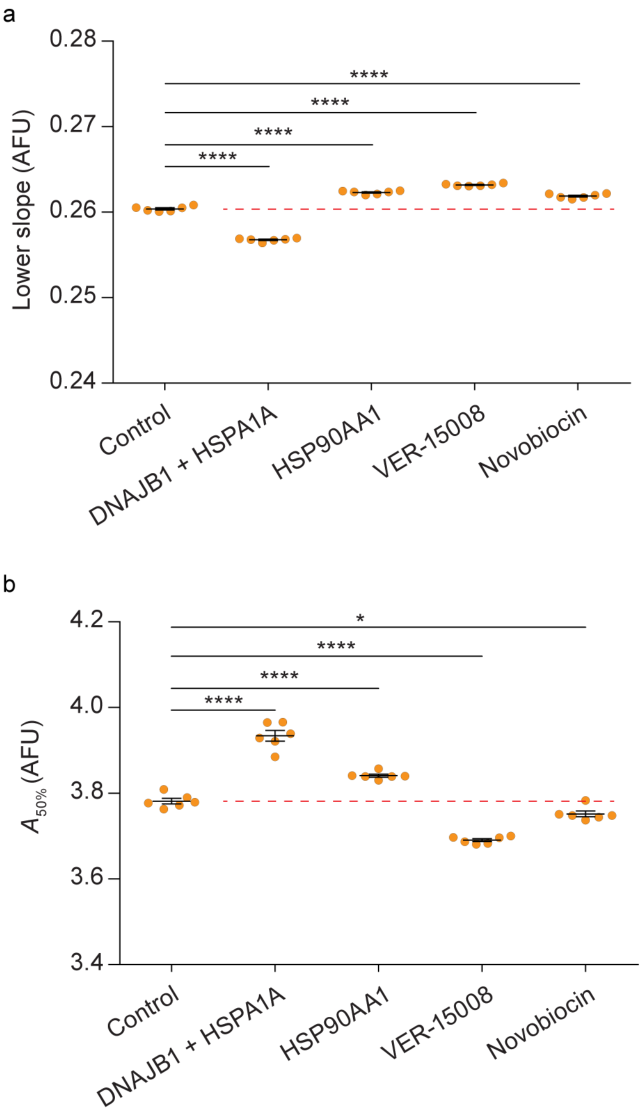


**Supplementary Fig. 6. Application of biosensor to probe proteostasis upon inhibition of HSP70 ATPase activity relative to other treatments.** HEK293 cells were transfected with I25A,I96G barnase biosensor. The transfections were performed so the same mass quantity of biosensor DNA was used for all treatments and cells were all analysed 48 h after transfection. Thus, control = ⅓ barnase + ⅔ invEM; DNAJB1 + HSPA1A = ⅓ barnase + ⅓ DNAJB1 + ⅓ HSPA1A; HSP90AA1 = ⅓ barnase + ⅔ HSP90AA1; VER-155008 = ⅓ barnase + ⅔ invEM + 20 µM VER-155008 for 18 hours; Novobiocin = ⅓ barnase + ⅔ invEM + 800 µM Novobiocin for 6 hours. **a**) Lower-slope analysis. **b**) $A_{50\%}$ analysis of the same treatment groups. Bars indicate means ± SEM. Results of a one way ANOVA with Dunnett's post-hoc test are coded as * $p<0.05$, ****$p<0.0001$. Red dashed lines are visual reference lines to the control means.

**Supplementary Fig. 7.  Full uncropped Western Blots.**

Fig 6a, IB HSF1

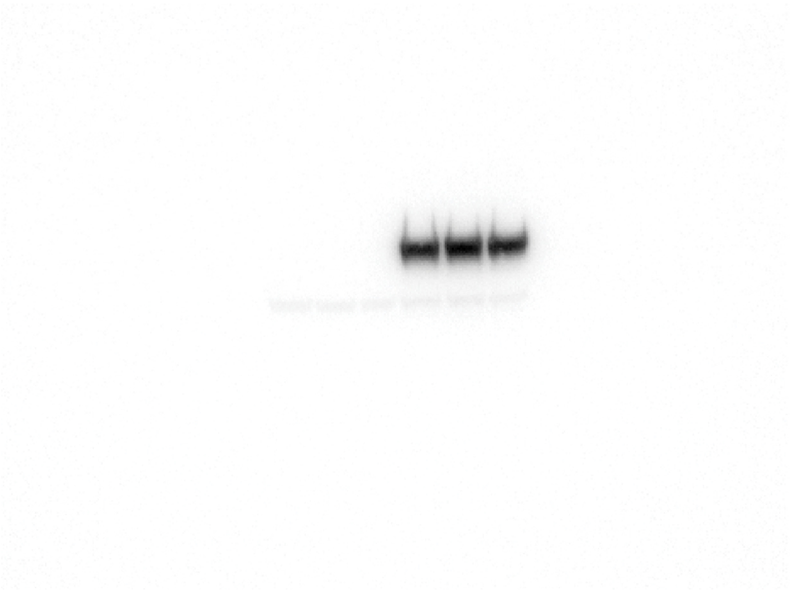

Fig 6a, IB Tubulin

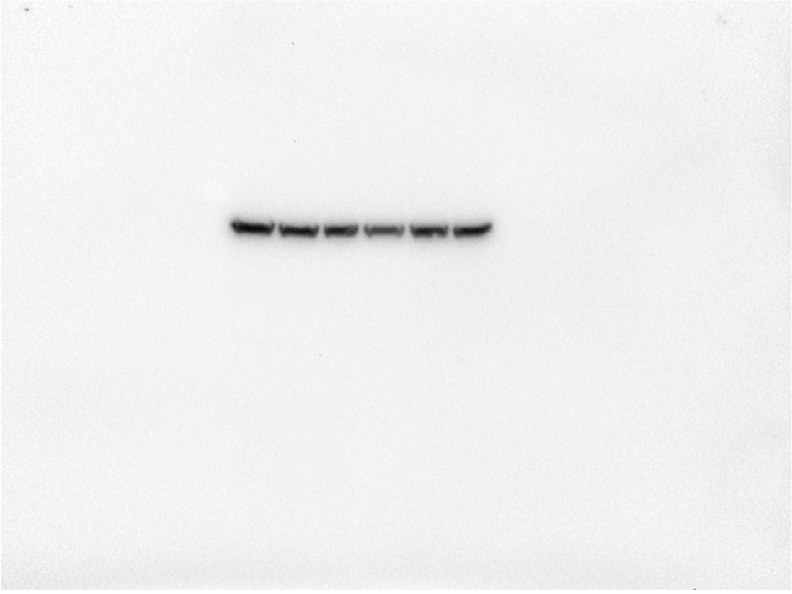

Fig 6a, IB myc-tag

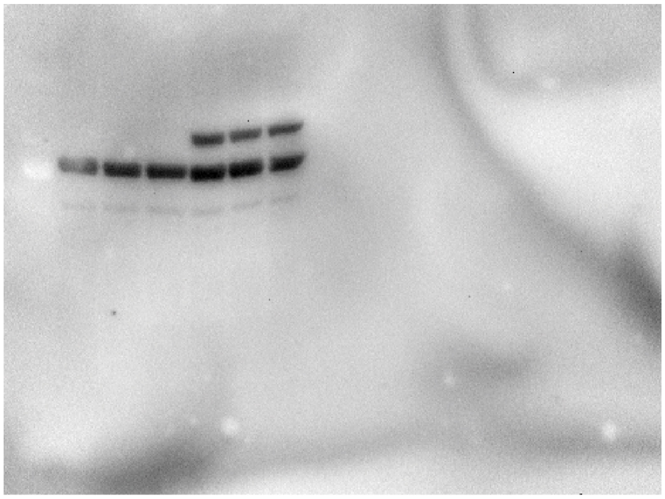

Fig 6a, IB Tubulin

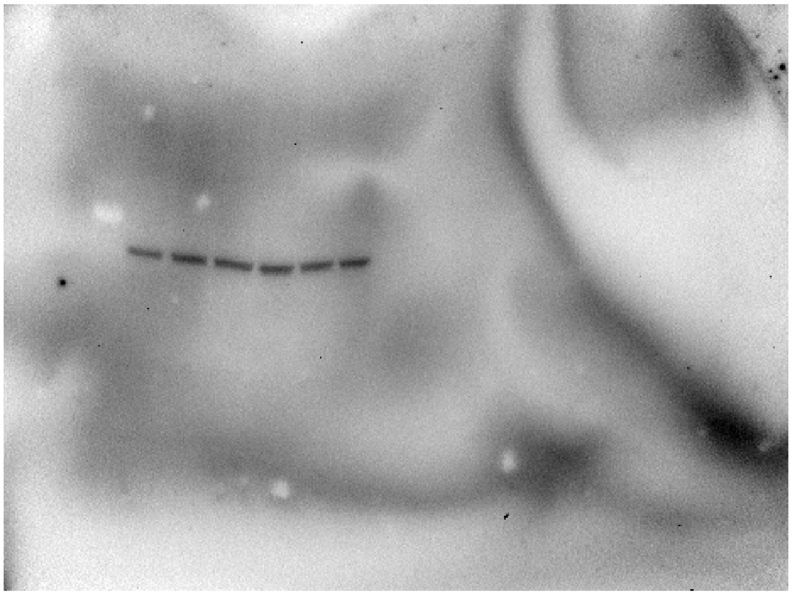

Fig 6b, IB: BAG1

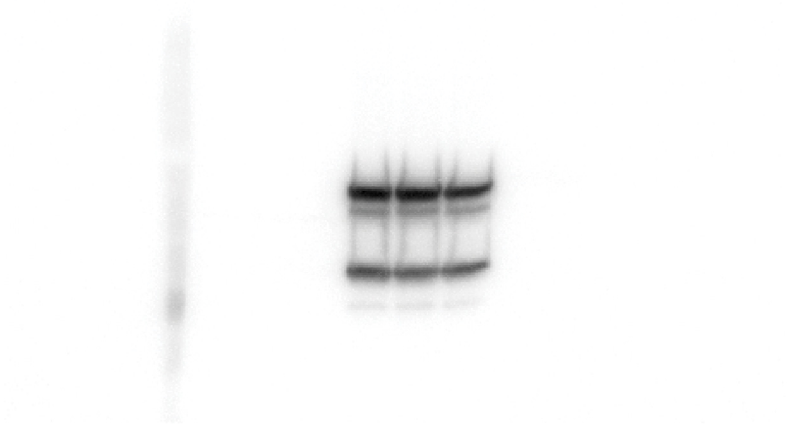

Fig 6b, IB Tubulin

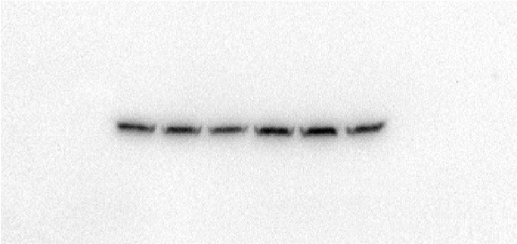

**Supplementary Table 1.** Free energies of folding of barnase fusion proteins, determined by FRET-based urea denaturation (in mammalian lysate)

| **Mutant*** | **Δ$G_F$ ± SD of three replicates (kJ/mol)** |
|---|---|
| wild-type* | –24.9 ± 2.3 |
| I55G | –18.7 ± 2.1 |
| V45T | –17.0 ± 1.9 |
| I25A | –13.0 ± 1.7 |
| I88A | –12.1 ± 1.8 |
| L14A | –11.7 ± 2.0 |
| I51A | –10.3 ± 2.0 |
| L89G | –9.6 ± 2.1 |
| I96G | –8.3 ± 1.7 |
| I55G L89G | –3.7 ± 1.3 |
| I88G | –3.2 ± 0.9 |
| V45T L89G | –2.5 ± 0.9 |
| I51A I88A | –1.4 ± 0.9 |
| V45T I88G | –0.7 ± 0.6 |
| I25A I96G | 0.8 ± 0.6 |

*All barnase constructs are based on the inactive H102A mutant (wild-type*) and fused to mTFP1 cp175 and Venus cp173

**Supplementary Table 2: Summary of proteins discovered by proteomics of barnase immunoprecipitants.**
Chaperones shown in **bold.** Ratios define enrichment for (I25A, I96G)/WT* barnase variants from HEK293T cells.

| Gene ID (Human) | Σ Coverage, % | Σ# Unique Peptides | Σ# PSMs | Normalised Abundance Ratio, Replicate I | Normalised Abundance Ratio, Replicate II | Normalised Abundance Ratio, Replicate III | Normalised Abundance Ratio, Replicate IV | Average Abundance Ratio | Abundance Ratio SD [%] | Score Mascot | Negative log10 p-value | Significance p-value<0.05 | emPAI |
|---|---|---|---|---|---|---|---|---|---|---|---|---|---|
| **HSPA1B** | **37.0** | **22** | **118** | **4.976** | **4.818** | **2.891** | **3.054** | **3.935** | **28.338** | **2508** | **1.873** | + | **64.432** |
| RUVBL1 | 14.9 | 6 | 28 | 3.810 | 3.145 | 2.079 | 1.605 | 2.660 | 37.660 | 802 | 1.345 | + | 3.217 |
| RUVBL2 | 10.6 | 4 | 16 | 2.721 | 2.585 | 1.541 | 1.382 | 2.057 | 34 | 512 | 1.256 | | 0.887 |
| **HSP90AB1** | **17.7** | **6** | **37** | **2.433** | **2.428** | **1.420** | **1.516** | **1.949** | **28.584** | **1153** | **1.375** | + | **1153** |
| **HSP90AA1** | **18.4** | **8** | **37** | **2.494** | **2.103** | **1.240** | **1.727** | **1.891** | **28.291** | **1120** | **1.350** | + | **1120** |
| **HSPA8** | **30.7** | **17** | **98** | **2.068** | **2.005** | **1.348** | **1.334** | **1.689** | **23.835** | **2647** | **1.380** | + | **2647** |
| TUBB | 14.2 | 2 | 26 | 2.134 | 1.261 | 1.152 | 0.848 | 1.349 | 41 | 777 | 0.529 | | 3.062 |
| RPL23 | 12.9 | 2 | 13 | 1.561 | 1.010 | 1.269 | 0.833 | 1.168 | 27 | 480 | 0.436 | | 2.162 |
| RS3 | 23.0 | 5 | 9 | 2.147 | 0.813 | 0.707 | 0.969 | 1.159 | 58 | 199 | 0.176 | | 1.336 |
| HSPA9 | 3.8 | 2 | 5 | 1.630 | 0.579 | 0.673 | 1.258 | 1.035 | 48 | 111 | 0.047 | | 0.233 |
| SFPQ | 4.2 | 2 | 6 | 1.178 | 0.900 | 0.939 | | 1.005 | 15 | 127 | 0.020 | | 0.403 |
| CCT3 | 3.9 | 2 | 6 | 1.213 | 0.915 | 0.805 | | 0.978 | 22 | 136 | 0.060 | | 0.199 |
| RPS16 | 21.9 | 4 | 17 | 1.047 | 0.833 | 0.762 | 0.330 | 0.743 | 41 | 293 | 0.730 | | 7.254 |
| RPS20 | 22.7 | 3 | 8 | 0.873 | 0.991 | 0.800 | 0.307 | 0.743 | 41 | 102 | 0.731 | | 3.642 |
| RPS13 | 11.3 | 2 | 3 | 0.493 | 0.427 | 0.924 | 0.542 | 0.597 | 37 | 84 | 1.439 | + | 0.874 |
| RPS11 | 18.4 | 4 | 12 | 0.687 | 0.400 | 0.891 | 0.378 | 0.589 | 42 | 232 | 1.355 | + | 5.31 |
| RPS25 | 14.4 | 2 | 8 | 0.699 | 0.246 | 0.616 | 0.314 | 0.469 | 47 | 187 | 1.759 | + | 3.642 |
| RPS14 | 39.7 | 4 | 16 | 0.795 | 0.354 | 0.474 | 0.237 | 0.465 | 52 | 147 | 1.676 | + | 18.307 |
| EDF1 | 23.6 | 3 | 8 | 0.624 | 0.426 | 0.366 | 0.282 | 0.425 | 34 | 126 | 2.377 | + | 1.61 |
| PRDX1 | 28.6 | 5 | 37 | 0.408 | 0.308 | 0.415 | 0.459 | 0.398 | 16 | 940 | 3.523 | + | 7.483 |
| RPL13 | 24.6 | 5 | 13 | 0.358 | 0.420 | 0.601 | 0.188 | 0.392 | 44 | 326 | 2.244 | + | 2.162 |
| SARNP | 27.1 | 5 | 12 | 0.569 | 0.361 | 0.403 | 0.214 | 0.387 | 38 | 338 | 2.456 | + | 3.924 |

| | | | | | | | | | | | | | |
|---|---|---|---|---|---|---|---|---|---|---|---|---|---|
| HDAC6 | 7.2 | 8 | 27 | 0.498 | 0.328 | 0.285 | 0.435 | 0.386 | 25.258 | 528 | 2.959 | + | 1.572 |
| RPL28 | 13.9 | 3 | 8 | 0.349 | 0.373 | 0.561 | 0.207 | 0.372 | 39 | 191 | 2.481 | + | 1.31 |
| NPM1 | 23.1 | 5 | 25 | 0.349 | 0.253 | 0.426 | 0.424 | 0.363 | 22 | 506 | 3.222 | + | 3.642 |
| HSD17B10 | 9.2 | 2 | 5 | 0.394 | 0.341 | 0.407 | 0.310 | 0.363 | 12 | 90 | 4.000 | + | 0.54 |
| RPS19 | 17.9 | 3 | 21 | 0.464 | 0.270 | 0.481 | 0.227 | 0.360 | 36 | 598 | 2.638 | + | 2.831 |
| STMN1 | 27.5 | 4 | 24 | 0.229 | 0.360 | 0.424 | 0.191 | 0.301 | 36 | 613 | 3.000 | + | 9 |
| SERBP1 | 16.4 | 7 | 40 | 0.335 | 0.283 | 0.367 | 0.197 | 0.296 | 25.151 | 581 | 3.523 | + | 7.254 |
| SNRPA1 | 9.0 | 2 | 3 | | 0.240 | 0.184 | 0.199 | 0.208 | 14 | 71 | 3.398 | + | 0.359 |

**Supplementary Table 3.** Nucleotide sequence of pTriEx mTFP1(cp175)-Barnase wild-type*-Venus(cp173)

tcctgcatcttttaatcaaatcccaagatgtgtataaacgcgccggtatgtacaggaagaggtttatactaaactgttacattgcaaacgtgg
tttcgtgtgccaagtgtgaaaaccgatgtttaatcaaggctctgacgcatttctacaaccacgactccaagtgtgtgggtgaagtcatgcat
cttttaatcaaatcccaagatgtgtataaaccaccaaactgccaaaaaatgaaaactgtcgacaagctctgtccgtttgctggcaactgcaa
gggtctcaatcctatttgtaattattgaataataaaacaattataaatgtcaaatttgttttttattaacgatacaaaccaaacgcaacaagaaca
tttgtagtattatctataattgaaaacgcgtagttataatcgctgaggtaatatttaaaatcattttcaaatgattcacagttaatttgcgacaatat
aattttattttcacataaactagacgccttgtcgtcttcttcttcgtattccttctctttttcatttttctcttcataaaaattaacatagttattatcgtat
ccatatatgtatctatcgtatagagtaaattttttgttgtcataaatatatatgtctttttttaatggggtgtatagtaccgctgcgcatagtttttctgt
aatttacaacagtgctattttctggtagttcttcggagtgtgttgctttaattattaaatttatataatcaatgaatttgggatcgtcggttttgtaca
atatgttgccggcatagtacgcagcttcttctagttcaattacaccatttttttagcagcaccggattaacataactttccaaaatgttgtacgaa
ccgttaaacaaaaacagttcacctcccttttctatactattgtctgcgagcagttgtttgttgttaaaaataacagccattgtaatgagacgcac
aaactaatatcacaaactggaaatgtctatcaatatatagttgctgatggccggcctattaatagtaatcaattacggggtcattagttcatag
cccatatatggagttccgcgttacataacttacggtaaatggcccgcctggctgaccgcccaacgacccccgcccattgacgtcaataat
gacgtatgttcccatagtaacgccaatagggactttccattgacgtcaatgggtggagtatttacggtaaactgcccacttggcagtacatc
aagtgtatcatatgccaagtccgccccctattgacgtcaatgacggtaaatggcccgcctggcattatgcccagtacatgaccttacggg
actttcctacttggcagtacatctacgtattagtcatcgctattaccatgctgatgcggttttggcagtacaccaatgggcgtggatagcggtt
tgactcacggggatttccaagtctccaccccattgacgtcaatgggagtttgttttggcaccaaaatcaacgggactttccaaaatgtcgta
ataaccccgccccgttgacgcaaatgggcggtaggcgtgtacggtgggaggtctatataagcagacgtcgtttagtgaaccgtcagatc
actagatgctttattgcggtagtttatcacagttaaattgctaacgccagtctcgaacttaacgtgcagaagttggtcgtgaggcactgggc
aggtaagtatcgggccctttgtgcgggggggagcggctcggggctgtccgcgggggggacggctgccttcgggggggacggggcag
ggcggggttcggcttctggcgtgtgaccggcggc**<u>tctaga</u>**gcctctgctaaccatgttcatgccttcttctttttcctacagctcctgggca
acgtgctggttattgtgctgtctcatcattttggcaaagaattggatcggaccgaaattaatacgactcactataggggaattgtgagcggat
aacaattccccggagttaatccgggacctttaattcaacccaacacaatatattatagttaaataagaattattatcaaatcatttgtatattaat
taaaatactatactgtaaattacattttatttacaatcaaaggagatatacc**atg**gcacaccatcaccaccaccacgggagcggggagca
gaaactaatatcagaggaggatcttggctcaggttcaggatccggcggccaccaccgcgttgacttcaagaccatctacagggccaag
aaggcggtgaagctgcccgactatcactttgtggaccaccgcatcgagatcctgaaccacgacaaggactacaacaaggtgaccgttt
acgagagcgccgtggcccgcaactccaccgacggcatggacgagctgtacaagggtgctagcggaggaatggtgagcaagggcg
aggagaccacaatgggcgtaatcaagcccgacatgaagatcaagctgaagatggagggcaacgtgaatggccacgccttcgtgatcg
agggcgagggcgagggcaagccctacgacggcaccaacaccatcaacctggaggtgaaggagggagccccctgcccttctccta
cgacattctgaccaccgcgttcgcctacggcaacagggccttcaccaagtaccccgacgacatccccaactacttcaagcagtccttcc
ccgagggctactcttgggagcgcaccatgaccttcgaggacaagggcatcgtgaaggtgaagtccgacatctccatggaggaggact
ccttcatctacgagatacacctcaagggcgagaacttcccccccaacggccccgtgatgcagaagaagaccaccggctgggacgcct
ccaccgagaggatgtacgtgcgcgacggcgtgctgaagggcgacgtcaagcacaagctgctgctggagggctccggggcccaagt
gatcaacaccttcgacggcgtggccgactacctgcagacctaccacaaactgcccgataactacatcaccaagagcgaggcccaggc
cctgggatgggtggcctctaagggaaacctggccgatgtggcccctggcaagtctatcggcggcgacatcttcagcaacagagaggg
aaagctgcctggcaagagcggcagaacttggagagaggccgacatcaactacaccagcggct**<u>tccgga</u>**acagcgacagaatcctgt
acagcagcgactggctgatctacaagacaaccgacgcctaccagaccttcaccaagatcagggcggccgcgatggacggcggcgtg
cagctcgccgaccactaccagcagaacacccccatcggcgacggccccgtgctgctgcccgacaaccactacctgagctaccagtcc
gccctgagcaaagaccccaacgagaagcgcgatcacatggtcctgctggagttcgtgaccgccgccgggatcactctcggcatggac
gagctgtacaagggtggcagcggtggcatggtgagcaagggcgaggagctgttcaccggggtggtgcccatcctggtcgagctgga
cggcgacgtaaacggccacaagttcagcgtgtccggcgagggcgagggcgatgccacctacggcaagctgaccctgaagctgatct
gcaccaccggcaagctgcccgtgccctggcccaccctcgtgaccaccctgggctacggcctgatgtgcttcgcccgctaccccgacc
acatgaagcagcacgacttcttcaagtccgccatgcccgaaggctacgtccaggagcgcaccatcttcttcaaggacgacggcaacta
caagacccgcgccgaggtgaagttcgagggcgacaccctggtgaaccgcatcgagctgaagggcatcgacttcaaggaggacggc

aacatcctggggcacaagctggagtacaactacaacagccacaacgtctatatcaccgccgacaagcagaagaacggcatcaaggcc
aacttcaagatccgccacaacatcgagggtaccgatatccttcaaaaaaaacttgaagaacttgaacttgacgaa**tga**gatatctaatgac
tcgagcaccaccatcaccatcaccatcactaagtgattaacctcaggtgcaggctgcctatcagaaggtggtggctggtgtggccaaaa
attatggggacatcatgaagccccttgagcatctgacttctggctaataaaggaaatttattttcattgcaatagtgtgttggaatttttgtgtc
tctcactcggaaggacatatgggagggcaaatcatttaaaacatcagaatgagtatttggtttagagtttggcaacatatgcccatatgtaa
ctagcataaccccttggggcctctaaacgggtcttgaggggttttttgctgaaagcatgcggaggaaattctccttgaagtttccctggtgtt
caaagtaaaggagtttgcaccagacgcacctctgttcactggtccggcgtattaaaacacgatacattgttattagtacatttattaagcgct
agattctgtgcgttgttgatttacagacaattgttgtacgtattttaataattcattaaatttataatctttagggtggtatgttagagcgaaaatca
aatgattttcagcgtctttatatctgaatttaaatattaaatcctcaatagatttgtaaaataggtttcgattagtttcaaacaagggttgtttttccg
aaccgatggctggactatctaatggattttcgctcaacgccacaaaacttgccaaatcttgtagcagcaatctagctttgtcgatattcgtttg
tgttttgttttgtaataaaggttcgacgtcgttcaaaatattatgcgcttttgtatttctttcatcactgtcgttagtgtacaattgactcgacgtaaa
cacgttaaatagagcttggacatatttaacatcgggcgtgttagctttattaggccgattatcgtcgtcgtcccaaccctcgtcgttagaagtt
gcttccgaagacgattttgccatagccacacgacgcctattaattgtgtcggctaacacgtccgcgatcaaatttgtagttgagctttttgga
attgcgatcgcataacttcgtatagcatacattatacgaagttataagctcggaacgctgcgctcggtcgttcggctgcggcgagcggtat
cagctcactcaaaggcggtaatacggttatccacagaatcaggggataacgcaggaaagaacatgtgagcaaaaggccagcaaaag
gccaggaaccgtaaaaaggccgcgttgctggcgtttttccataggctccgcccccctgacgagcatcacaaaaatcgacgctcaagtca
gaggtggcgaaacccgacaggactataaagataccaggcgtttccccctggaagctccctcgtgcgctctcctgttccgaccctgccgc
ttaccggatacctgtccgcctttctcccttcgggaagcgtggcgctttctcaatgctcacgctgtaggtatctcagttcggtgtaggtcgttc
gctccaagctgggctgtgtgcacgaaccccccgttcagcccgaccgctgcgccttatccggtaactatcgtcttgagtccaacccggtaa
gacacgacttatcgccactggcagcagccactggtaacaggattagcagagcgaggtatgtaggcggtgctacagagttcttgaagtg
gtggcctaactacggctacactagaagaacagtatttggtatctgcgctctgctgaagccagttaccttcggaaaaagagttggtagctctt
gatccggcaaacaaaccaccgctggtagcggtggtttttttgtttgcaagcagcagattacgcgcagaaaaaaaggatctcaagaagat
cctttgttaccaatgcttaatcagtgaggcacctatctcagcgatctgtctatttcgttcatccatagttgcctgactccccgtcgtgtagataa
ctacgatacgggagggcttaccatctggccccagtgctgcaatgataccgcgagacccacgctcaccggctccagatttatcagcaata
aaccagccagccggaagggccgagcgcagaagtggtcctgcaactttatccgcctccatccagtctattaattgttgccgggaagctag
agtaagtagttcgccagttaatagtttgcgcaacgttgttgccattgctacaggcatcgtggtgtcacgctcgtcgtttggtatggcttcattc
agctccggttcccaacgatcaaggcgagttacatgatcccccatgttgtgcaaaaaagcggttagctccttcggtcctccgatcgttgtca
gaagtaagttggccgcagtgttatcactcatggttatggcagcactgcataattctcttactgtcatgccatccgtaagatgcttttctgtgact
ggtgagtactcaaccaagtcattctgagaatagtgtatgcggcgaccgagttgctcttgcccggcgtcaatacgggataataccgcgcca
catagcagaactttaaaagtgctcatcattggaaaacgttcttcggggcgaaaactctcaaggatcttaccgctgttgagatccagttcgat
gtaacccactcgtgcacccaactgatcttcagcatcttttactttcaccagcgtttctgggtgagcaaaaacaggaaggcaaaatgccgca
aaaaagggaataagggcgacacggaaatgttgaatactcatactcttcctttttcaatattattgaagcatttatcagggttattgtctcatgtc
cgcgcgtt

**Key**

**Start codon**
mTFP1 cp175
Barnase wild-type* (H102A)
Venus cp173
**Stop codon**
BspEI/XmaI ligation site –used to insert barnase
NotI – used to insert barnase
**XbaI, BspEI** – used to isolate single barnase mutants from double-mutant constructs

**Supplementary Note 1: Further discussion of the assumptions underlying our classification of Lower-slope and Upper-slope populations.**

In our analysis of the Upper-slope data, we assume that these cells simply have a higher FRET population arising from extensive intermolecular FRET. This may provide insight to how aggregates assemble in the cell – but we stress that we do not analyse the FRET data from aggregates quantitatively for this purpose in our study. It is important to note that it is not mechanistically insightful to understand the correlation between the $\Delta G$ and the Upper-slope. This is because the relative FRET efficiency (which is measured by the gradient, or slope) can only be mechanistically understood in the intramolecular FRET context (i.e. in cells with soluble biosensor; i.e. the lower FRET slope). In cells with aggregates, FRET will dominate from intermolecular interactions. This will lead to an overall much higher FRET state than the soluble biosensor (i.e. the fundamental basis we use to separate cells with aggregates from those without).

While we don't expect the values of the high FRET state to vary between barnase mutants since they are all likely to form the same (or similar) aggregate structures, there does appear to be differences among the mutants as shown below (Fig. SN1-1).

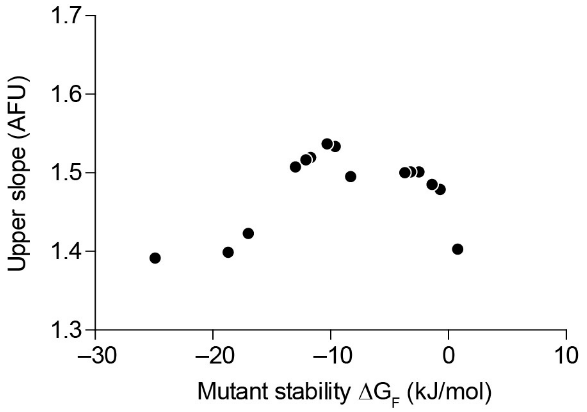


**Figure SN1-1**: Upper-slope values of barnase biosensor mutants under baseline conditions (in HEK293T cells).

It appears that as the biosensor becomes more unfolded the aggregates display a higher FRET signal, which may be explained by the far greater tendency to aggregate inside the cells to enable much higher FRET signal.

Another question we pondered was whether $\Delta A$ really reflects different aggregate loads. To the best approximation the data suggests this is the case. Our analysis works on the basis that aggregation is highly cooperative in cells – i.e. cells fall into binary populations of those with aggregates and those without – this is supported visually by microscopy data (Fig. 2c) and also from other studies investigating aggregation-prone proteins [4-6]. Visual inspection of cells collected by sorting on the flow cytometer validate the high fidelity of cells allocated to the *Lower* and Upper-slope populations (Fig. SN1-2).

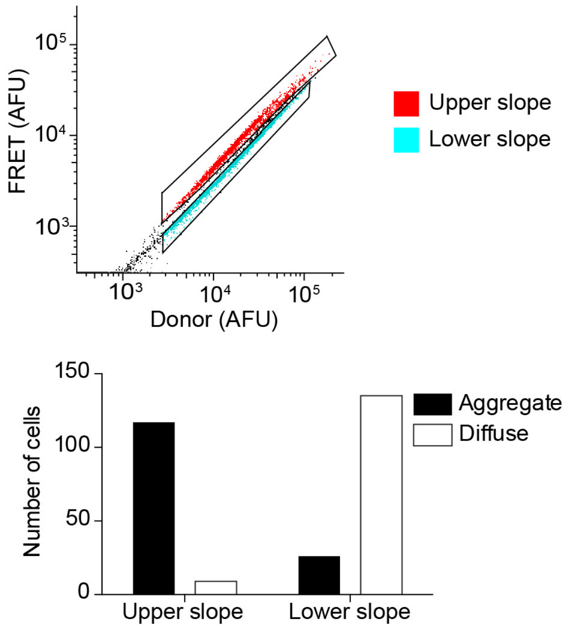


**Figure SN1-2**: Visual inspection of HEK293 cells transfected with the I25A, I96G barnase variant. Cells were recovered by a sorting flow cytometer and manually checked for visible aggregate content (*lower graph*).

While we cannot rule out the accumulation of diffuse submicroscopic aggregates in the Lower-slope population, given that the FRET signal closely matches the expected foldedness from Δ*G*, as shown in the manuscript (Fig. 3c) then distortions arising from minor contributions of oligomers would at best have negligible influence on the data.

**Supplementary Note 2: Derivation of Equation 1.**

To derive Equation 1, we defined the following: $K_d$ is the binding affinity of chaperone to unfolded barnase, $K_f$ is the folding equilibrium constant of barnase, *B* is the total concentration of barnase in the cell, [*U*] is the concentration of free unfolded barnase, *u* is the fraction of barnase in this state, [*F*] is the concentration of native (folded) barnase, *f* is the fraction of barnase in this state, [*UH*] is the concentration of unfolded barnase in complex with chaperone, *h* is the fraction of barnase in this state, [*H*] is the concentration of free chaperone, and *C* is the latent chaperone concentration (i.e. the sum of [*H*] and [*UH*]).

Equations S1 – S4 are derived from the definitions above.

$$K_d = \frac{[U].[H]}{[UH]} \quad \text{Equation S1}$$

$$K_f = \frac{[F]}{[U]} \quad \text{Equation S2}$$

$$[F]+[U]+[UH]=B$$ Equation S3

$$[H]+[UH]=C$$ Equation S4

$[F]=f.B$ Equation S5.1 $[U]=u.B$ Equation S5.2 $[UH]=h.B$ Equation S5.3

Rearrange (S4) and substitute into (S1)

$$K_d=\frac{[U].(C-[UH])}{[UH]}$$ Equation S6

Substitute (S5.1-S5.3) into (S6)

$$K_d=\frac{u.(C-h.B)}{h}$$

$$K_d=u.\left(\frac{C}{h}-B\right)$$ Equation S7

Substitute (S5.1-S5.2) into (S2)

$$K_f=\frac{f}{u}$$

$$u=\frac{f}{K_f}$$ Equation S8

Substitute (S5.1-S5.3) into (S3)

$$f+u+h=1$$ Equation S9

Substitute (S8) into (S9)

$$f+\frac{f}{K_f}+h=1$$

$$h=1-f.\left(1+\frac{1}{K_f}\right)$$ Equation S10

Substitute (S8) and (S10) into (S7)

$$K_d = \frac{f}{K_f}\left(\frac{C}{1 - f.\left(1 + \frac{1}{K_f}\right)} - B\right)$$

$$C = \left[\frac{K_d.K_f}{f} + B\right].\left[1 - f.\left(1 + \frac{1}{K_f}\right)\right] \qquad \text{Equation S11}$$

To derive Equation 1, we defined $C_c$ and $f_c$ as the latent chaperone concentration and barnase fraction folded, respectively, under control conditions. $C_t$ and $f_t$ are the latent chaperone concentration and barnase fraction folded under treatment conditions.

$$\Delta C = C_c - C_t$$

$$\Delta C = \left[\frac{K_d.K_f}{f_c} + B\right].\left[1 - f_c.\left(1 + \frac{1}{K_f}\right)\right] - \left[\frac{K_d.K_f}{f_t} + B\right].\left[1 - f_t.\left(1 + \frac{1}{K_f}\right)\right]$$

$$\Delta C = \frac{K_d.K_f}{f_c} - f_c.B\left(1 + \frac{1}{K_f}\right) - \frac{K_d.K_f}{f_t} + f_t.B\left(1 + \frac{1}{K_f}\right)$$

$$\Delta C = \frac{-K_d.K_f.(f_c - f_t)}{f_c.f_c} - (f_c - f).B\left(1 + \frac{1}{K_f}\right) \qquad \text{Equation 1}$$

**Supplementary Note 3: Modeling the effect of proteostasis on the balance between folded and unfolded barnase.**

To best connect our data to the model we (i) defined a single barnase concentration (i.e. $B$) for each dataset, (ii) corrected data for intrinsic fluorescence changes, (iii) derived fraction folded ($f$) from the Lower-slope gradients, (iv) defined a suitable value for the averaged binding affinity $K_d$, then (v) applied Equation 1. This supplementary note describes these processes in detail to guide readers through the data analysis steps.

i. **Define barnase concentration ($B$)**
Calculation of $\Delta C$ (Equation 1) is dependent on the cellular concentration of barnase (i.e. $B$). To define a single value of $B$ for each dataset, cells classified as belonging to the Lower-slope population (see materials and methods) were analyzed for Venus acceptor fluorescence and restricted to a range between the background signal and 0.2 of the maximum fluorescence signal (which was about 560-4,600 AFU in our instrument settings). This concentration range provided a trade-off between having low barnase concentration at which the model predicts chaperone engagement will be most pronounced, having enough cells for high quality data and having sufficient signal above background to accurately determine the Lower-slope gradient.

To define $B$ in standard units, we converted the flow cytometry arbitrary fluorescence units (AFU) to micromolar (µM). A sample of cells expressing Venus cp173 was analyzed by flow cytometry and plate reader in parallel. Plate reader fluorescence intensities of suspensions with known cell densities were calibrated to a standard curve of purified Venus cp173. The cell volume was measured by confocal z-stacks (3,000 $\mu m^3 \pm$ 1,700 $\mu m^3$, mean ± standard deviation of 19 cells) to enable estimation of the average cellular Venus cp173 concentration from plate reader data. Comparing this to the average flow cytometry fluorescence intensity yielded the conversion factor of 9.8 AFU $\mu M^{-1}$. Thus, an average barnase concentration $B$ = 265 µM was used in all calculations.

ii. **Correct data for intrinsic fluorescence changes**
To address the possibility that certain treatments could change the fluorescence properties of the donor and acceptor fluorophores via changes in the cellular environment, we used a FRET-positive control comprising the donor and acceptor fluorophores fused via a short linker (denoted mTFP1-Venus) in all experiments. Under each condition (treatment or control), the Lower-slope gradient of each barnase mutant, as well as mTFP1-Venus, was determined by linear regression using the low-concentration cell subset described above. The proportional change in Lower-slope gradient of mTFP1-Venus, under treatment conditions compared to control, was used as a correction factor to account for changes in the barnase constructs that could be attributed to fluorophore-dependent changes. For example, chaperone overexpression reduced the Lower-slope gradient of mTFP1-Venus by 0.7% compared to the EGFP Y66L

control (i.e. to 99.3%, or 0.993) (Fig. 3b). Therefore, the Lower-slope gradient of each barnase dataset was corrected for intrinsic fluorescence changes by dividing by 0.993.

**iii. Derive fraction folded (*f*) from the Lower-slope gradients**

Deriving fraction folded ($f$) from the Lower-slope gradients required estimating folded and unfolded "baselines", that is, Lower-slope gradients that would correspond to barnase that was 100% folded and 100% unfolded, respectively. We used the Lower-slope gradients under control conditions to estimate folded and unfolded baselines by the best fit to their thermodynamically defined folding equilibrium positions (Fig. 3c). The corrected Lower-slope gradients were then scaled against these folded and unfolded baselines to calculate $f$. This approximation enabled unbiased estimation of folded and unfolded baselines from the data, but artificially constrained the control data to values that assume no influence from proteostasis and therefore risked overestimating $f$. To minimize error arising from inaccuracy in $f$, we calculated the change in $C$ upon chaperone overexpression (i.e. $\Delta C$, Equation 1), which is very sensitive to changes in $f$ ($f_t – f_c$, i.e. the difference in fraction folded of a given barnase mutant between treatment and control conditions) but quite robust to small error in $f$.

**iv. Define a suitable $K_d$**

While there are likely large numbers of different chaperones and other quality control proteins able to bind to barnase, we can approximate the net effect of these interactions with a single averaged binding affinity ($K_d$). HSP70 binding affinities have been reported for various substrates, and generally fall in the low micromolar range [7]. Rather than predict $K_d$ a priori, the chaperone binding affinity was estimated from chaperone overexpression data using the method of least squares to minimize variation between $\Delta C$ estimates calculated from different mutants of barnase. The best-fit $K_d$ of 2.9 µM, lay in the expected range and was used in all other experiments.

**v. Apply Equation 1**

A single value of $\Delta C$ was then calculated for each mutant under each treatment. The values of $B$=265 µM $K_d$=2.9 µM applied to all barnase variants, whereas $K_f=e^{-\Delta G/RT}$ was specific to each mutant and was calculated from experimentally measured $\Delta G_F$ values (Fig. 1c, Supplementary Table 1). For each treatment, $f_t$ and $f_c$ were averaged across three replicates and used to calculate ($f_t$ - $f_c$). Since the denominator of the first term in Equation 1 (i.e. $f_t.f_c$) was most sensitive to error in the estimation of $f$, we applied an upper limit to these values of the theoretical maximum $f$ value (i.e. the fraction folded defined by the folding equilibrium constant in the absence of chaperone engagement, $f_{max}=1/(1+1/K_f)$).

As $f$ approaches its theoretical maximum, the signal disappears as the second term in Equation 1 goes to zero, and when coupled with high $K_f$ values, this leads to sensitivity and noise. We

therefore excluded the most stable barnase mutants (wild-type*, V45T, and I55G) from our analysis. The reported $\Delta C$ values were therefore averaged across 12 mutants of barnase.

**Supplementary References:**


1 Serrano, L., Kellis, J. T., Jr., Cann, P., Matouschek, A. & Fersht, A. R. The folding of an enzyme. II. Substructure of barnase and the contribution of different interactions to protein stability. *J. Mol. Biol.* **224**, 783-804, doi:10.1016/0022-2836(92)90562-x (1992).

2 Vu, N. D., Feng, H. & Bai, Y. The folding pathway of barnase: the rate-limiting transition state and a hidden intermediate under native conditions. *Biochemistry (Mosc).* **43**, 3346-3356, doi:10.1021/bi0362267 (2004).

3 Danielsson, J. *et al.* Thermodynamics of protein destabilization in live cells. *Proc. Natl. Acad. Sci. U. S. A.* **112**, 12402-12407, doi:10.1073/pnas.1511308112 (2015).

4 Polling, S. *et al.* Misfolded polyglutamine, polyalanine, and superoxide dismutase 1 aggregate via distinct pathways in the cell. *J. Biol. Chem.* **289**, 6669-6680, doi:10.1074/jbc.M113.520189 (2014).

5 Polling, S. *et al.* Polyalanine expansions drive a shift into alpha-helical clusters without amyloid-fibril formation. *Nat Struct Mol Biol* **22**, 1008-1015, doi:10.1038/nsmb.3127 (2015).

6 Hipp, M. S. *et al.* Indirect inhibition of 26S proteasome activity in a cellular model of Huntington's disease. *J. Cell Biol.* **196**, 573-587, doi:10.1083/jcb.201110093 (2012).

7 Mayer, M. P. *et al.* Multistep mechanism of substrate binding determines chaperone activity of Hsp70. *Nat. Struct. Biol.* **7**, 586-593, doi:10.1038/76819 (2000).
