# Supplementary material for: A biosensor-based framework to measure latent proteostasis capacity
Source: Nat Commun. 2018 Jan 18;9:287. doi: 10.1038/s41467-017-02562-5 (PMC5773518; doi:10.1038/s41467-017-02562-5)
Supplement: Supplementary file 2 — Description of Additional Supplementary Files [file 41467_2017_2562_MOESM2_ESM.pdf]

Description of Additional Supplementary Files

File Name: Supplementary Data 1
Description: Lower-slope values in selected figures used to calculate the ΔC
